# Gene Expression Profiling of Immune-Competent Human Cells Exposed to Engineered Zinc Oxide or Titanium Dioxide Nanoparticles

**DOI:** 10.1371/journal.pone.0068415

**Published:** 2013-07-22

**Authors:** Soile Tuomela, Reija Autio, Tina Buerki-Thurnherr, Osman Arslan, Andrea Kunzmann, Britta Andersson-Willman, Peter Wick, Sanjay Mathur, Annika Scheynius, Harald F. Krug, Bengt Fadeel, Riitta Lahesmaa

**Affiliations:** 1 Turku Centre for Biotechnology, University of Turku and Åbo Akademi University, Turku, Finland; 2 Turku Doctoral Programme of Biomedical Sciences, Turku, Finland; 3 Department of Signal Processing, Tampere University of Technology, Tampere, Finland; 4 Swiss Federal Laboratories for Material Science and Technology, Laboratory for Materials-Biology Interactions, St. Gallen, Switzerland; 5 Inorganic and Materials Chemistry, University of Cologne, Cologne, Germany; 6 Division of Molecular Toxicology, Institute of Environmental Medicine, Karolinska Institutet, Stockholm, Sweden; 7 Translational Immunology Unit, Department of Medicine Solna, Karolinska Institutet and University Hospital, Stockholm, Sweden; National Institute of Health (NIH), United States of America

## Abstract

A comprehensive *in vitro* assessment of two commercial metal oxide nanoparticles, TiO_2_ and ZnO, was performed using human monocyte-derived macrophages (HMDM), monocyte-derived dendritic cells (MDDC), and Jurkat T cell leukemia-derived cell line. TiO_2_ nanoparticles were found to be non-toxic whereas ZnO nanoparticles caused dose-dependent cell death. Subsequently, global gene expression profiling was performed to identify transcriptional response underlying the cytotoxicity caused by ZnO nanoparticles. Analysis was done with doses 1 µg/ml and 10 µg/ml after 6 and 24 h of exposure. Interestingly, 2703 genes were significantly differentially expressed in HMDM upon exposure to 10 µg/ml ZnO nanoparticles, while in MDDCs only 12 genes were affected. In Jurkat cells, 980 genes were differentially expressed. It is noteworthy that only the gene expression of metallothioneins was upregulated in all the three cell types and a notable proportion of the genes were regulated in a cell type-specific manner. Gene ontology analysis revealed that the top biological processes disturbed in HMDM and Jurkat cells were regulating cell death and growth. In addition, genes controlling immune system development were affected. Using a panel of modified ZnO nanoparticles, we obtained an additional support that the cellular response to ZnO nanoparticles is largely dependent on particle dissolution and show that the ligand used to modify ZnO nanoparticles modulates Zn^2+^ leaching. Overall, the study provides an extensive resource of transcriptional markers for mediating ZnO nanoparticle-induced toxicity for further mechanistic studies, and demonstrates the value of assessing nanoparticle responses through a combined transcriptomics and bioinformatics approach.

## Introduction

Nanomaterials are in size comparable to biological structures [1]. The small size enables nanoparticles to be introduced in the biological systems via cellular uptake and their interaction with internal or membrane molecules. Nanomaterials have a high carrier capacity and because of their size, they can pass cellular barriers making them potent carriers of drugs and other small molecules. Thus nanotechnology holds promises for broad variety of new biological and biochemical applications. On the other hand, the large reactive surface area of nanomaterials is thought to cause more severe adverse effects on organisms than microscale materials. Thus, in-depth analysis of the cellular responses to nanomaterials is needed before they can be safely used. We have taken a step toward this direction by characterizing in detail the transcriptional changes caused by the commercial ZnO and TiO_2_ engineered nanoparticles (EN).

Metal oxide nanoparticles are produced and used in large amounts in consumer products such as sunscreens. At the same time, common awareness of possible negative effects of chemicals has raised public concern. This has led to an urgent need for careful risk assessment of nanoparticles and consecutively generation of objective information of possible unfavorable effects. There have been a number of studies showing evidence of adverse effects of TiO_2_ and ZnO-ENs in different cellular systems. On the other hand, it has been pointed out that there are limitations regarding the conclusions or extrapolation of some of the results to the human health [2], [3]. In several occasions, the nanoparticles used have not been satisfactorily characterized or the experimental conditions are not reported in detail. However, careful design and documentation is an essential basis for valuable interpretation of the nanoparticle studies [4], [5]. Titanium dioxide is very insoluble and thermally stable. It cannot pass undamaged skin, and even when inhaled or ingested TiO_2_ is not thought to have serious effects on humans. However, there are also reports indicating that TiO_2_ particles may be considered as a biohazard. For instance, pulmonary exposure of mice to respirable-size TiO_2_ during pregnancy has been shown to increase a risk of asthma susceptibility in the offspring [6]. ZnO-ENs release Zn^2+^ ions, which are known to cause cytotoxicity [7]–[9]. In addition, ZnO nanoparticle-specific effects have been reported [10]–[13]. In the experimental setup used in the present study, ZnO-EN toxicity is known to be primarily mediated by released Zn^2+^ ions [7]. Hence our aim was to further identify, which genes respond to ZnO-EN exposure. In general, imbalance of zinc ions can have deleterious effects to cellular homeostasis because even as high proportion as 10% of human proteins are predicted to bind zinc thereby representing the most abundant class of metalloproteins. Zinc is an especially important trace metal for transcription factors and almost half of them need this ion for proper function [14], [15].

In the present study, the gene expression of human cells exposed to ZnO and TiO_2_ nanoparticles was analyzed with microarrays to elucidate how these materials modulate transcription in different cell types. Comprehensive bioinformatics analyses were conducted to classify gene signatures and discern patterns of EN-induced transcriptional regulation. Immune-competent cells i.e. macrophages and dendritic cells were selected because the immune system is the first line of defense against foreign intrusion and a detailed understanding of nanomaterial effects on the immune system is critically important [16], [17]. The human Jurkat T cell leukemic cell line, a commonly used immune cell line in toxicological research, was included to explicate whether different cell types respond discordantly or similarly to metal oxide nanoparticle exposure. We also compared toxicity and global transcriptional response derived from an exposure of Jurkat cells with a panel of chemically and physically distinct ZnO-ENs.

Microarray platform provides means for analysis of thousands of genes at the same time. In our study, Illumina's Sentrix HumanHT-12 arrays and Affymetrix GeneChip Human Genome U219 array plates were used, both of which contain probes covering the whole human genome. The approach used in the study presents a guideline at which level new nanomaterials should be analyzed; the materials used should be well-characterized with chemical, physical and toxicity assays in a variety of cell types. Microarray analysis is a powerful method for identification of regulated genes and pathways, leading to generation of novel hypothesis. As the expense of the array technology has decreased, transcriptomics analysis offers a suitable target-gene-wise unbiased analysis method reporting an important level of gene expression regulation. The need for such a high-throughput method is well-recognized, and it is expected to become an important and wide-spread tool for the characterization of EN effects on biological systems [12], [18].

In summary, we have performed a detailed toxicogenomic characterization of the transcriptional changes caused by commercially obtained ZnO and TiO_2_ nanoparticles. Our study provides a number of candidate genes and signaling pathways, which may be mediating ZnO-EN derived cellular toxicity. In addition, the results obtained with a panel of modified ZnO nanoparticles, support that the cellular response to ZnO nanoparticles is caused by particle dissolution. In contrast, we show that TiO_2_-EN does not lead to changes in gene expression after short *in vitro* exposure supporting the view that TiO_2_ is not toxic *per se*. Overall, the results can be used as a resource of transcriptional markers to ZnO-EN exposure in HMDM, MDDC and Jurkat cells, and may serve to increase our understanding of the underlying toxicity mechanisms in different cellular systems.

## Results and Discussion

### Nanomaterial synthesis and characterization

Altogether nine ZnO and one TiO_2_ ENs were used to analyze their cellular effects (**Table 1**). The ZnO ENs used in the experiments can be classified according to their particle morphology, surface modification and size (**Table 1**, **Figure S1, S2 and S3**). Commercially available ZnO-1 nanoparticles synthesized with flame pyrolysis were modified with mandelic acid or covered with a silica shell and mercaptopropyl trimethoxy silane arm increasing the water solubility to produce ZnO-2 and ZnO-3, respectively [7]. TEM analysis revealed that ZnO-1 EN was highly polydispersive. Thus, the extremely big and small nanoparticles were excluded in the calculation of the particle size distribution. The average size of ZnO-1 was 14.7±1.07 nm and a diameter of 11.7±0.77 nm was acquired for ZnO-2. The core size distribution of ZnO-3 (14.6±1.2 nm core with 10.9±0.65 nm shell) was almost identical to ZnO-1 confirming the conducted particle counting. ZnO-4 (7.8±0.88 nm) and ZnO-5 (5.4±0.5 nm) are methoxyl and diethylene glycol modified ENs synthesized by a modified solvothermal method [7], [19] and a microwave method, respectively. Attached organic molecules can be detected by Fourier transform infrared spectroscopy (FT-IR) at 1411 cm^−1^ and at 667 cm^−1^ for ZnO-5 (**Table S1**). ZnO-6 was produced by thermal decomposition of the molecular precursor Zn(Oleate)_2_. After the synthesis, mandelic acid was used in the surface modification in the solvent based conditions to imitate ZnO-2 resulting in EN with a size of 13.2±0.6 nm. Correspondingly, mandelic acid functionalization was detected by FT-IR with the peaks at 1592 cm^−1^ and 1405 cm^−1^ similarly as with ZnO-2 [7]. High amount of weight loss in the thermogravimetric analysis (TGA) was also noticeable. ZnO-7, ZnO-8 and ZnO-9 are organic acid ligand covered nanoparticles with different sizes. ZnO-7 has been obtained by basic condition reflux of the Zn(Oleate)_2_ and phase transfer of the resulted oleate capped ZnO particles. Particle size analysis revealed 4.89±0.54 nm average diameter and noticeable agglomeration, which is due to the highly polar –OH side groups of the gluconic acid protective ligand as evidenced with FT-IR peaks at 3370 cm^−1^, 1598 cm^−1^ and 1428 cm^−1^. Citric acid provided water solubility for the ZnO-8 particles obtained from thermal decomposition of Zn(Oleate)_2_ having a triangle-like morphology and particle size of 34.5±2.4 nm. Folic acid modified ZnO-9 was obtained by varying the thermal decomposition conditions to achieve an elongated rod shape for ZnO with an average diameter of 40.4±2.6 nm and length of 404±8 nm. Provided water solubility was confirmed by FT-IR analysis detecting the peaks at 1582 cm^−1^ and 1410 cm^−1^ for ZnO-8, and 1617 cm^−1^ and 1563 cm^−1^ for ZnO-9.

**Table 1:** Physicochemical characterization of the ENs.

EN	modification	TEM size (nm)	DLS size (nm) Water/Medium	PDI Water/ Medium	Zeta potential Water/Medium
**ZnO-1** a	-	14.7±1.07	159±3/153±4	0.15/0.12	-23.9±0.2/-17.3±0.1
**ZnO-2** b	mandelic acid	11.7±0.77	312±3/287±2	0.10/0.12	-9.9±0.2/-12.3±0.2
**ZnO-3** b	mercaptopropyl-trimethoxysilane	14.6±1.2 (core), 10.9±0.65 (shell)	548±4/341±5	0.14/0.14	-22.4±0.6/-23.3±0.4
**ZnO-4**	methoxyl	7.8±0.88	387±1/110±4	0.14/0.15	-17.9±0.2/-12.7±0.4
**ZnO-5**	diethylene glycol	5.4±0.5	125±4/278±3	0.12/0.11	19.2±0.2/13.6±0.3
**ZnO-6**	mandelic acid	13.2±0.6	83±1/194±5	0.12/0.13	-9.4±0.3/-11.2±0.1
**ZnO-7**	gluconic acid	4.89±0.54	256±6/355±3	0.08/0.12	24.3±0.5/12.6±0.2
**ZnO-8**	citric acid	34.5±2.4	576±5/331±4	0.11/0.13	-5.9±0.4/-14.1±0.6
**ZnO-9**	folic acid	404±8 (length), 40.4±2.6 (diameter)	412±4/466±3	0.15/0.15	-31.7±0.3/-15.4±0.5
**TiO_2_** c	-	31.3±3.9	297±2/340±3	0.12/0.13	-14.3±0.3/-12.2±0.1

a IBU-tec advanced materials AG.

b Modified ZnO-1.

c Evonik Degussa (Aeroxide® TiO2 p25).

ZnO-5 to ZnO-9 EN-production is described in Methods S1. The corresponding data for ZnO-2 to ZnO-4 is shown in Buerki-Thurnherr *et al*. [7].

Dynamic light scattering (DLS), polydispersity index (PDI) and Zeta potential data for all particles after 3 h of storage is shown in Table S2.

The data is an average of three measurements with the standard deviations.

X-ray diffraction (XRD) analysis confirmed that all the ZnO particles form hexagonal wuertzite crystals (**Table S1**) [7]. XRD spectra also unveiled the purity of the ENs, and were in line with the TEM analysis of EN size as calculated by the Scherrer formula (data not shown). Dynamic light scattering (DLS) was used to determine the hydrodynamic size of the dispersed ENs both in pure water and in the cell culture medium (**Table 1**). DLS sizes were significantly larger than the primary sizes for all the ENs indicating that they form aggregates in aqueous solutions. ZnO-3, ZnO-4 and ZnO-8 form smaller aggregates in the presence of the cell culture medium components than in pure water. Instead, agglomeration of ZnO-5, ZnO-6, ZnO-7 and ZnO-9 increases in the cell culture medium. Zeta potentials of all ENs indicated that the ZnO-EN dispersions are not stabilized by electrostatic repulsion and they are prone to form aggregates (**Table 1**). Based on low polydispersity indexes (PDI<0.2) all ENs form relatively uniform aggregates, characteristic for each EN (**Table 1**). Neither Zeta potential, PDI nor DLS results changed when EN suspensions were stored for 3 h at room temperature (22±2°C) before the measurement indicating that each EN acquired its characteristic dispersion pattern immediately after preparation of the samples (**Table S2**).

### Toxicity and dissolution of ZnO nanoparticles

**Figure 1:**
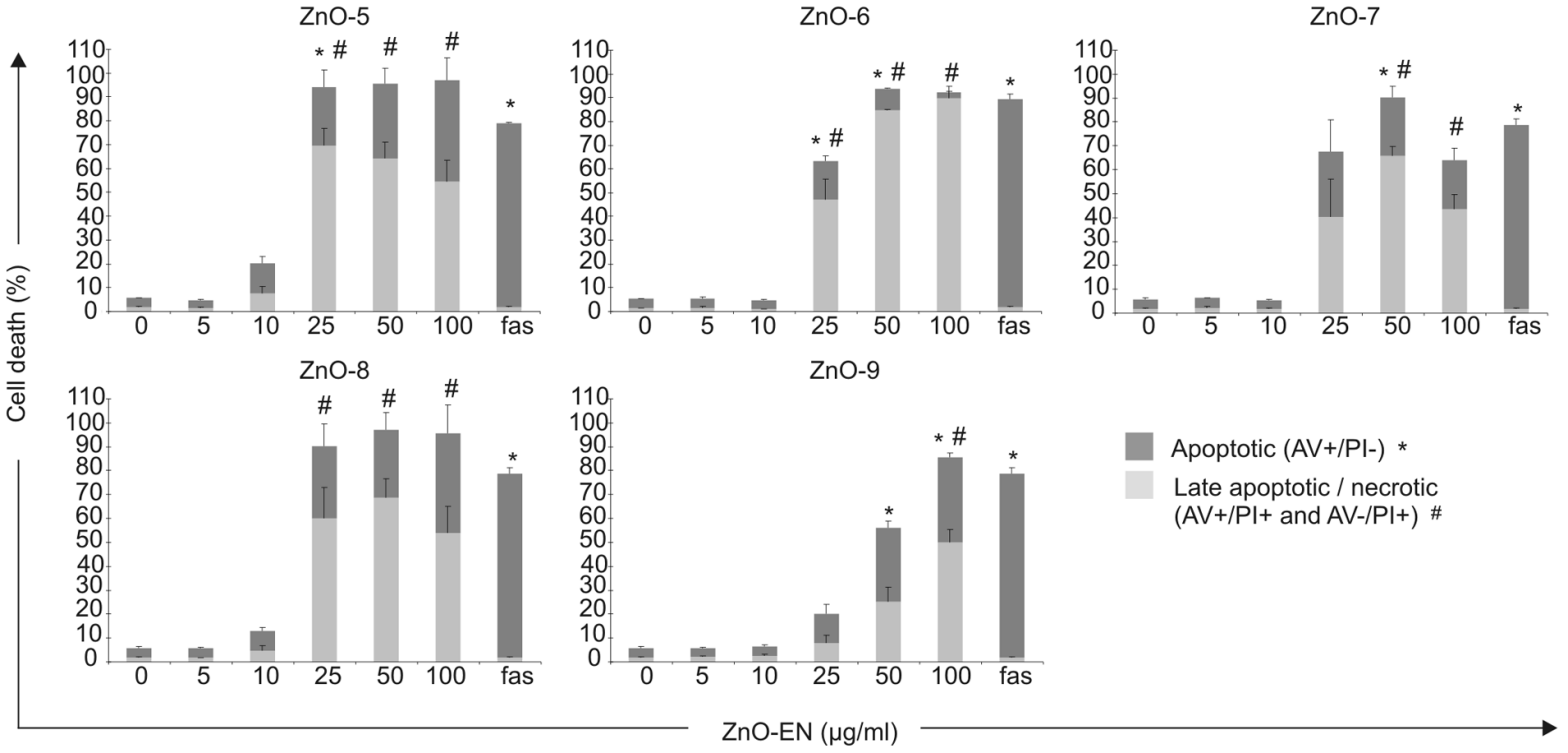
Cell viability data with different doses of ZnO-5, ZnO-6, ZnO-7, ZnO-8 and ZnO-9. Significance of the results has been determined with Student's t-test. Statistically significant differences (p<0.05) and standard errors of mean are indicated in the figure. The data is an average of three independent cultures. The protocol for propidium iodide (PI) and Annexin V (AV) staining, and the viability data for ZnO-1 to ZnO-4 is reported in Buerki-Thurnherr *et at*. [7] Fas ligand (fas) was used as a positive control in the analysis.

We have previously shown that the extracellular dissolution of ZnO-1, ZnO-2, ZnO-3 and ZnO-4 particles largely determines their toxicity [7]. To further analyze this interdependency, we investigated the toxicity of ZnO-5, ZnO-6, ZnO-7, ZnO-8 and ZnO-9 to immortalized Jurkat T-cells (**Figure 1**). In a dose-response assay ZnO-5 turned out to be the most toxic and ZnO-9 the least toxic of the analyzed ZnO ENs (**Figure 1**). Dissolution kinetics of all particles were analyzed by attentive separation of the soluble and precipitated fractions with three consecutive centrifugations for each sample after 30 min, 6 and 24 h of incubation followed by determination of the amount of Zn^2+^ in aqueous phase with flame atomic absorption spectroscopy (F-AAS). Results revealed that the particles did not reach the dissolution equilibrium before 6 h, instead the dissolution increased linearly over the timepoints (minimum R^2^: ZnO-1 (0.81), maximum R^2^: ZnO-7 (0.99)) (**Table 2**).

**Table 2:** Dissolution kinetics of ZnO-EN suspensions in the cell culture medium and their toxicity to Jurkat cells at 24 h.

EN	Ligand type	30 min (mg/liter)/RSD%	6 h (mg/liter)/RSD%	24 h (mg/liter)/RSD%	cell death^a^ 10 µg/ml	cell death^a^ 25 µg/ml
**ZnO-1**	Unknown	0.34/0.50	1.85/0.30	2.65/0.20	32^b^	98^b^
**ZnO-3**	Core-shell	0.60/1.40	0.62/1.20	1.29/0.20	7^b^	13^b^
**ZnO-4**	Ether bonding	1.04/0.20	1.10/1.20	1.81/0.10	7^b^	7^b^
**ZnO-5**	Ether bonding	0.84/0.10	1.29/0.10	2.16/0.10	20	94
**ZnO-2**	Carboxylic acid	1.42/0.00	1.82/0.10	2.44/0.60	6^b^	58^b^
**ZnO-6**	Carboxylic acid	1.21/0.40	1.32/0.00	2.17/0.40	5	63
**ZnO-7**	Carboxylic acid	0.90/0.10	1.24/0.10	2.16/0.30	5	68
**ZnO-8**	Carboxylic acid	1.28/0.20	1.44/0.30	3.62/0.60	13	90
**ZnO-9**	Carboxylic acid	0.67/0.40	0.69/0.30	1.33/0.30	6	20

a Apoptotic + late apoptotic/necrotic cells.

b Buerki-Thurnherr *et al*. 2013 [7].

The data is an average of three measurements.

Cell death and the amount of released Zn^2+^ did not directly correlate. For example, citric acid modified ZnO-8 is not the most toxic of the ENs as could be expected based on the dissolution data alone. However, cell death and the amount of released Zn^2+^ showed synthesis and surface ligand type dependent correlation (**Table 2**). It is clearly seen that surface modification modulates dissolution and free Zn^2+^ amount, which is playing a role in cell toxicity. Nevertheless, we cannot exclude the possibility that there still is some nanoparticle-specific component in the response. TGA and FT-IR analysis (**Table S1**) revealed that the core of the commercial ZnO-1 synthesized by flame pyrolysis is covered with some unknown organic compounds as highlighted above. Mercaptopropyl-trimethoxylanesilane core/shell coating of ZnO-1 provides relatively durable and water-soluble protection for ZnO-3 leading to low toxicity. The ligand coverage of ZnO-4 and ZnO-5 is achieved with Zn-O-C metal-ether bonding of methoxyl and diethylene glycol, respectively. TEM images of these two particles showed high agglomeration (**Figure S2**). In addition, ZnO-5 has an organic shell of diethylene glycol groups on the particle surface, which may affect the Zn^2+^ release properties in the aqueous conditions. The Zn^2+^ dissolution and toxicity of ZnO-4 and ZnO-5 are directly correlated. ZnO-2, ZnO-6, ZnO-7, ZnO-8 and ZnO-9 have all carboxylic acid ligand on their surface for water solubility and surface modulation. Their dissolution follows the pKa order of the organic acid used to modify the particle surface (**Table 2**). Hence Zn^2+^ dissolution can be interpreted with the agitation of the ZnO-EN surface by these relatively strong carboxylic acid ligands [20]. When particles are introduced into aqueous phase, spontaneous formation of hydroxides increases the removal of surface ligands, and the interaction of the ligand and Zn^2+^ causing further release of Zn^2+^ from the surface of ZnO EN. However, ZnO-2 releases more Zn^2+^ than expected based on its toxicity. This is most probably due to the surface chemistry of ZnO-1 obtained by flame pyrolysis which may vary the final dissolution of ZnO-2. F-AAS reveals the total amount of soluble Zn^2+^ released, but not the complexes they form nor their impact on cell death, which can be modulated with particle modification. Thus, the relative contribution of Zn^2+^ complexes on cellular toxicity may vary according to the aqueous environment surrounding the ZnO ENs. Our results showed that ZnO nanoparticle solubility and toxicity can be programed with pre-designed modification of the particle surface by functional ligands and their attachment type.

### Effects of TiO_2_ and ZnO-1 ENs on HMDM, MDDC and Jurkat cell transcriptome

Based on our earlier mechanistic data on ZnO-1, we know that its toxicity is largely caused by the extracellular release of Zn^2+^ ions in the experimental conditions used. It is also produced in large quantities and found in many consumer products. Thus it was selected along with commercial TiO_2_ nanoparticles to the genome-wide transcriptional profiling (**Figure S4, Table S3**). Three cell types were studied; Jurkat, an immortalized T-cell line widely used in laboratories as well as primary human monocyte-derived dendritic cells (MDDC) and monocyte-derived macrophages (HMDM) as models of primary cells likely to interact with nanoparticles upon exposure. The doses of 1 µg/ml and 10 µg/ml were selected based on cell viability analysis (**Figure S5**) to represent an exposure when there was not any detectable toxicity (1 µg/ml) and on the other hand a sub-lethal dose (10 µg/ml) putatively revealing a strong but also a specific transcriptional response not related solely to overt cell death. All three cell types responded similarly to the nanoparticles; TiO_2_-ENs were apparently non-toxic whereas dose-dependent cytotoxicity was observed for ZnO-1 (**Figure S5**). The timepoints 6 and 24 h were chosen to represent immediate and accumulative early nanoparticle exposure effects on gene expression. Illumina Sentrix HumanHT-12 Expression BeadChips were used to analyze the global gene expression in the samples from three replicate experiments (**Figure S4, Table S3**). First, robust analysis of the data was done to reveal the general trends of the responses. This crude analysis (**Figure S6**), which was based only on the magnitude of the average gene expression change over the replicates, showed that TiO_2_ particles were very inert. This correlated well with the cell viability results and the general view on the reactivity of TiO_2_ nanoparticles with cell types and tissues. In contrast to our result, TiO_2_ causes cell death to mouse bone marrow derived dendritic cells also after short *in vitro* exposures with the same 10 µg/ml dose as used in our study [21]. The discrepancy may be due to different composition of the TiO_2_ particles used, different cellular origin of the cells, or variation in TiO_2_ sensitivity between the organisms. Our study aimed at analyzing only the short-term effects of TiO_2_ nanoparticles *in vitro* at subtoxic concentrations so the effects of continuous exposure are not evident and cannot be predicted based on the current results.

The ZnO-1 nanoparticles caused both up- and downregulation of genes in the target cells (**Figure S6**). There was a dose response to ZnO-1 in all the cell types as the lower dose of 1 µg/ml regulated only a fraction of the number of genes regulated with the higher 10 µg/ml dose. To find out the most reliable markers of ZnO-EN exposure, we used a *limma* statistical analysis [22] to detect the differentially expressed genes. Probes with false discovery rate (FDR) less than 0.05 and absolute fold change (FC) at least 1.5 were selected for further analysis. By using these thresholds there were no differentially expressed genes after TiO_2_-EN or 1 µg/ml ZnO-1 treatment in any of the cell types or timepoints. In previous microarray analysis of colon epithelial carcinoma cell lines, CaCo-2 and RKO, sublethal dose of TiO_2_-EN was shown to have a specific, although limited, repressive transcriptional response [10]. However, the follow-up study including also melanocyte SK Me1–28 and keratinocyte HaCaT cell lines showed that TiO_2_-EN treated cells clustered together with the control cells [10].

**Figure 2:**
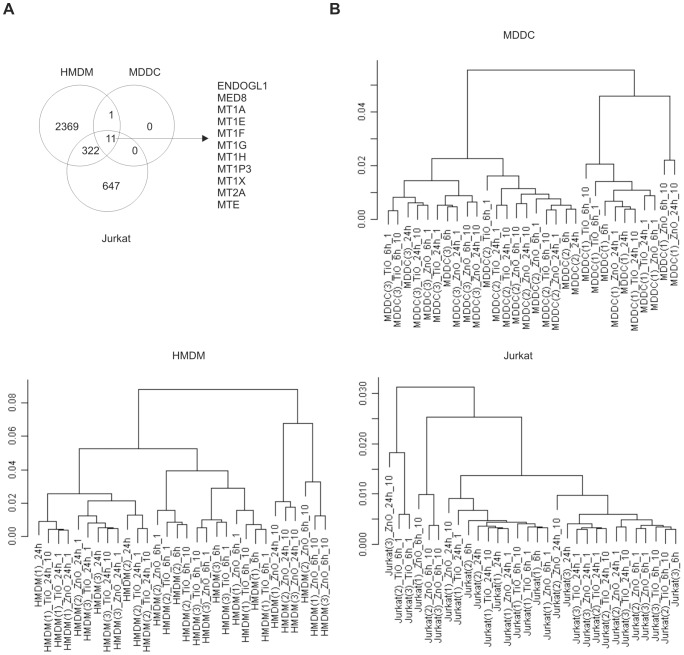
ZnO-1 and TiO_2_ induced gene expression in HMDM, MDDC and Jurkat cells. A) Venn diagram of the genes regulated in the each cell type within 24 h of exposure to 10 µg/ml of ZnO-1 nanoparticle. The numbers in the figure represent the genes detected with the probes filtered with the cut-off criteria FDR≤0.05 and absolute FC≥1.5 in comparison to untreated control cells. The genes regulated in each cell type are listed in the figure. B) Hierarchical clustering of the ZnO-1 or TiO_2_ treated, or untreated control samples in each cell type using correlation distance and complete linkage in clustering. Three replicate Jurkat cell cultures, or MDDCs or HMDMs from three different donors were used in the analysis. The gene expressions were measured with Illumina Sentrix HumanHT-12 Expression BeadChips. The name of the sample is formed by combining the information about cell type, number of replicate in brackets, treatment, timepoint and dose (µg/ml).

The differentially expressed probes after 10 µg/ml ZnO-1 exposure correspond to 2703, 980 and 12 differentially expressed genes in HMDM, Jurkat and MDDC samples, respectively (**Figure 2A** **, Table S4**). Cell viability of MDDC, HMDM and Jurkat cells after 24 h of exposure with 10 µg/ml of ZnO-1 was around 70%, 55% and 70% respectively [7], [23]. Although the cell viability of MDDC and Jurkat cells is at the same level, the transcriptional response varies substantially, showing that these outcomes do not directly correlate in all cell types. The number of regulated genes in MDDC samples is significantly lower than in HMDM or Jurkat cells. In hierarchical clustering the HMDM 10 µg/ml ZnO-1 samples separate into their own branch (**Figure 2B**), as most of the Jurkat samples. However instead, the individuals, not the treatments determine the clustering of the MDDC samples.

### Comparison of the transcriptional response of HMDM, MDDC and Jurkat cells to ZnO-1 exposure

**Figure 3:**
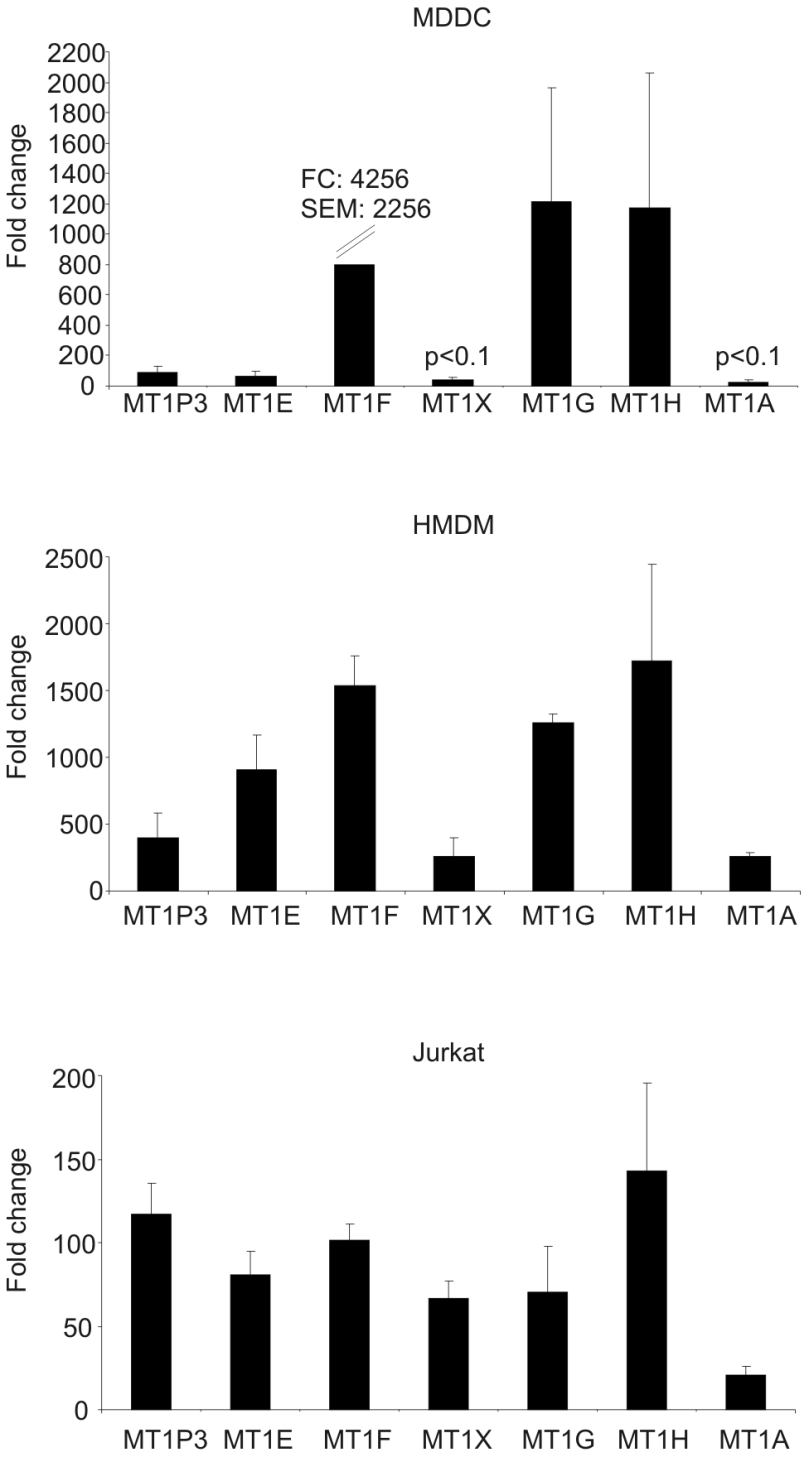
RT-PCR validation of the upregulation of metallothionein genes. MDDC, HMDM and Jurkat cells were exposed to ZnO-1 nanoparticles (10 µg/ml) and analyzed at 24 h timepoint. All the differences are statistically significant with Student's t-test p-value <0.05 if not otherwise indicated. Fold changes have been calculated against the corresponding untreated control sample, and it represents the average of three individuals or replicate cultures. When the expression level of the gene of interest was under the detection limit in the control sample, Ct-value was arbitrarily set to 35. The total number of RT-PCR cycles run was 40. Ct-values were normalized against housekeeping gene *EF1α* before the fold changes were calculated. Data is an average of three replicate cultures. Standard errors of mean are indicated in the figure.

Most of the genes regulated by 10 µg/ml of ZnO-1 in all three cell types studied belong to the family of metallothioneins (**Figure 2A**, **Figure S7**). The highly inducible expression of these genes was validated with independent sample sets with RT-PCR (**Figure 3** **, Table S3**). Strong induction of metallothioneins was also validated in MDDC samples, which in general did not get strongly activated by ZnO-1 exposure based on the transcriptional profiling. The commercial ZnO-1 nanoparticles used in this study undergo rapid dissolution in cell culture medium [7] and increase of free zinc ions is known to induce upregulation of metallothionein [10], [24], [25]. Metallothioneins are a heterogeneous family of low-molecular weight cysteine-rich proteins, which bind metal ions. They regulate cellular metal ion homeostasis and detoxification, and protection against oxidative stress. Metallothioneins have several isoforms which have both overlapping and specific roles [26]. Interestingly, knockout studies have shown that metallothionein is needed for phagocytosis and antigen-presentation by macrophages. The knockout mice have also impaired production of proinflammatory cytokines such as IL1β and IL6 [27]. *MTF1*, the most studied mediator of metal ion regulated transcription was upregulated in ZnO-1-exposed HMDM and Jurkat cells. In contrary to our finding, previous reports have shown that *MTF1* transcription is induced after zinc depletion in a THP-1 cell line [28] and downregulated in prostate cancer PC-3 cells after zinc exposure [29].

**Figure 4:**
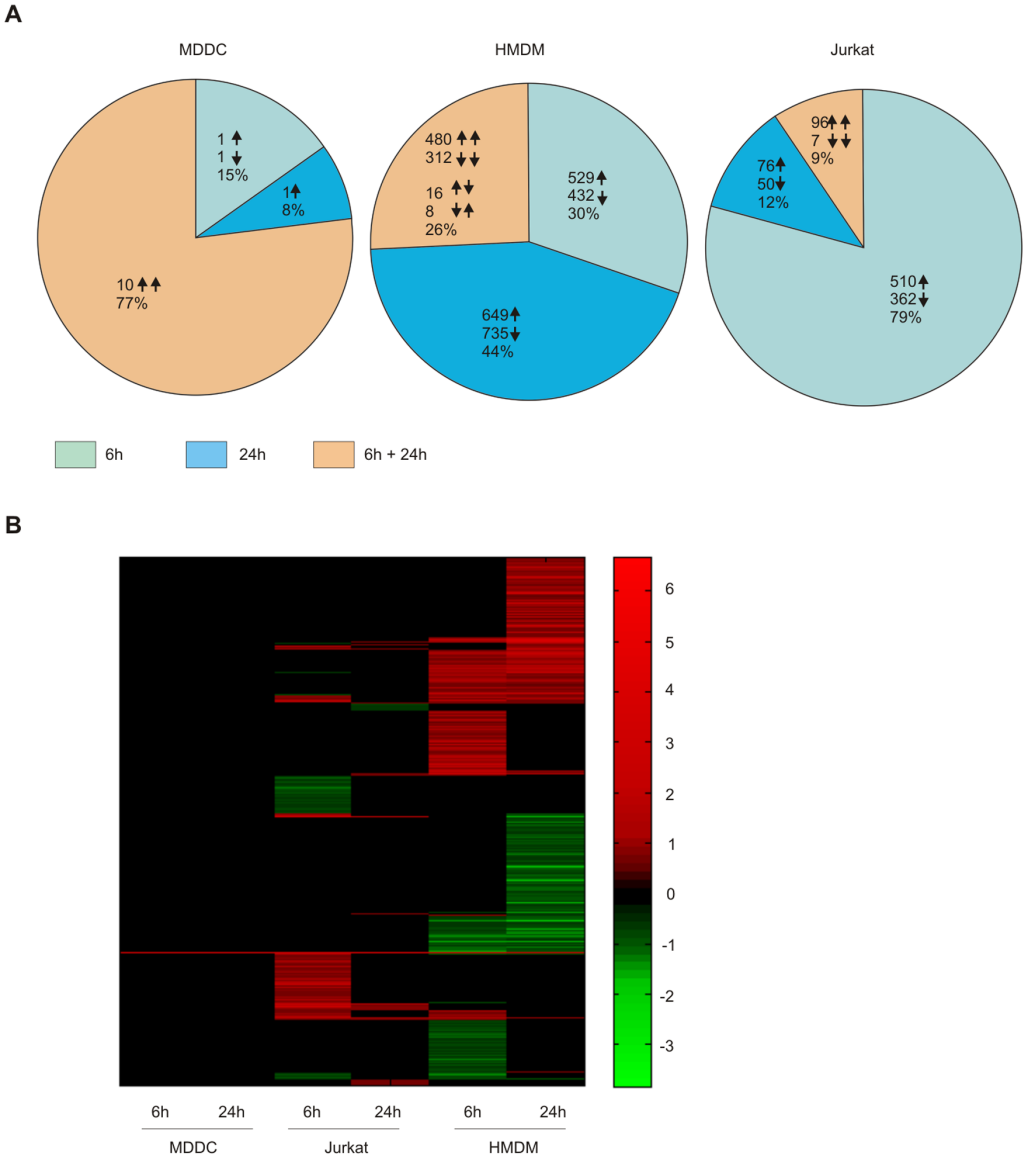
Comparison of ZnO-1 derived transcriptional response in MDDC, HMDM and Jurkat cells. A) The statistically significant differences in comparison between 10 µg/ml ZnO-1 nanoparticle treated cells and the untreated control cells. The numbers in the figure represent the probes with the cut-off criteria FDR≤0.05 and absolute fold change ≥1.5. The Illumina Sentrix HumanHT-12 Expression BeadChip probes have been categorized based on the kinetics of the differential expression. The arrows indicate the number of up and downregulated probes in ZnO-1 treated cells. In a segment of probes regulated at both timepoints, the arrows indicate the direction of the regulation at specific timepoint. The first arrow corresponds to the 6 h and the second the 24 h response. B) Heatmap of the all fold changes between the ZnO-1 treated and non-treated samples. The gene is colored only if the differential expression was statistically significant (FDR≤0.05 and absolute FC≥1.5). All the other, not significant, fold change-values are changed to black. Data is from three replicate cultures.

**Figure 5:**
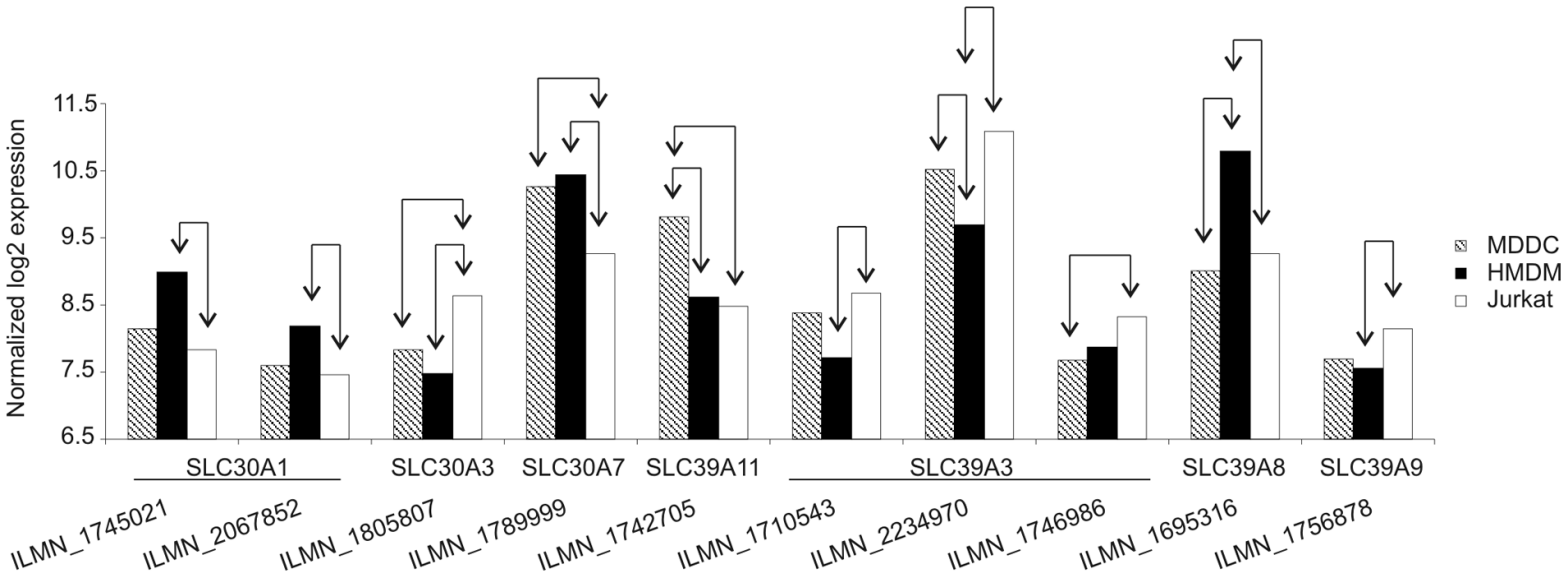
Differential expression of zinc transporters in MDDC, HMDM and Jurkat cells. The expression of SLC30 and SLC39 family members was analyzed in untreated Jurkat, MDDC and HMDM at 6 h timepoint. The expression of the genes, which showed differential expression among the cell types studied, is shown in the figure. The statistically significant comparisons in Illumina Sentrix HumanHT-12 Expression BeadChip data are marked with arrows (FDR≤0.05, absolute FC≥1.5). Data is an average of three replicate cultures.

As some genes are measured with more than one probe in the array, the number of differentially expressed probes is higher than the number of differentially expressed genes (**Figure 2A**). Altogether, we found 13 probes to be regulated in MDDC, 3161 in HMDM and 1101 in Jurkat cells (**Figure 4A** **, Table S4**). The kinetics of the ZnO-1-induced response varied between the cell types. Overall, the Jurkat cells tend to respond fast to ZnO-1 nanoparticle exposure. Most of the differentially expressed probes were found after 6 h of exposure. In contrast, there were proportionally more differentially expressed probes in HMDM after 24 h of exposure. In HMDM samples 24 probes detected both at 6 h and 24 h had an opposite expression pattern between the measurement timepoints. In contrary, in MDDC and Jurkat cells the expression pattern of the differentially expressed probes detected at 6 h remained the same at 24 h. The kinetic fashion of ZnO-EN induced cell stress and toxicity related transcription has previously been reported a study using a murine alveolar epithelial cell line [30] and recently confirmed in a high-throughput screen of the Keio *E. coli* knockout clone library [12]. Yet, our study further revealed that the time-dependency of the response is cell-type specific. Although common ZnO-1 induced changes were detected in all three cell types, most of the gene expression changes found were cell type-specific (**Figure 4B**). There were surprisingly few genes responding in a similar fashion to ZnO-1 even between primary HMDM and MDDC samples. Our previous results with Jurkat cells show that ZnO-1 release zinc ions extracellularly and Zn^2+^ ions enter the exposed cells [7]. The different transcriptional response to elevated zinc levels between the cell types studied, suggests that these cell types vary in their sensitivity to zinc. As all the cells were cultured in buffered culturing mediums, changes in pH should not regulate the zinc ion influx or sensing. Mammalian zinc homeostasis is regulated by transporters of SLC39 and SLC30 families, which increase or decrease the cytoplasmic zinc concentration, respectively [31]. Previous studies have shown that leukocyte subsets and cell lines regulate different zinc exporters in response to variations in zinc concentration. Of all zinc exporters, SLC30A1 was shown to be the most highly expressed in PBMCs under physiological conditions [32]. Indeed, we found upregulation of zinc exporters *SLC30A1* and *SLC30A2* both in HMDM and Jurkat cells after ZnO-1 treatment. HMDM upregulated also *SLC30A3* and *SLC39A8* in response to ZnO-1 exposure. In addition to changes in intracellular zinc levels via ion transport, zinc has been shown to regulate gene expression via binding to a specific receptor, GPR39 [33]. The expression of *GPR39* was at the same level among the untreated cells analyzed in our study (data not shown), so it does not explain the differential sensitivity of MDDC, HMDM and Jurkat cells to ZnO-1. Instead, we found that the expression of *SLC30A1*, *SLC30A3*, *SLC30A7*, *SLC39A3*, *SLC39A8*, *SLC39A9* and *SLC39A11* varies between the cell types studied (**Figure 5** **, Table S5**). Our results, and the previous findings with other zinc transporters [34], suggest that different cell types have distinct susceptibility to variations of extracellular zinc. This underlines the need of careful and wide characterization of each EN in general, because based on our data the cell type-specific responses can conceal the general gene expression patterns.

The genes regulated by ZnO-1 in HMDM and Jurkat cells at different timepoints were classified based on their function with Ingenuity Pathway Analysis software (www.ingenuity.com). Percentage of genes belonging to each category, proportion of up and downregulated genes in each category, and enrichment of functional classes over the genes present on the array was calculated (**Table 3**). There was a statistically significant enrichment of cytokines among the genes regulated in HMDM, and most of the genes belonging to this category were upregulated at both timepoints. Similarly, transcripts coding transmembrane receptors were enriched at 6 and 24 h timepoints. Phosphatases were also found to be enriched among the regulated genes at 6 h. In Jurkat cells, there was an enrichment of transcription regulators and kinases at 6 and 24 h timepoints, respectively. Interestingly though, preferential upregulation of kinases was found at 6 h and transcriptional regulators at 24 h. In general, the analysis suggested that there are differences both in the molecules driving the ZnO-1 induced gene response, and the functional outcome between these two cell types. In addition, regulation of kinases and phophatases indicated that posttranslational regulatory mechanisms are activated after ZnO-1 exposure. To capture the impact of these mechanisms to ZnO-1 derived toxicity, use of protein level analysis methods such as phosphoproteomics would be valuable.

**Table 3:** The number and percentage of the differentially expressed genes belonging to different functional classes based on the Ingenuity knowledge base.

	Number of genes				Percentage of genes			
	HMDM		Jurkat		HMDM		Jurkat	
	6h	24h	6h	24h	6h	24h	6h	24h
**Cytokine**	34a,b	33a,b	4	1	2a,b	2a,b	0	0
**Enzyme**	280	357	156	26	19	20	18	13
**G-protein coupled receptor**	21	28	8	0	1	2	1	0
**Growth factor**	11	13	5	3	1^b^	1	1	1
**Ion channel**	14	32	6	3	1	2	1	1
**Kinase**	79	77	32^b^	17^a^	5	4	4^b^	8^a^
**Ligand-dependent nuclear receptor**	6	4	1	0	0	0	0	0
**Other**	722	883	462	119	48	48	54	57
**Peptidase**	27	55	11	2	2	3	1	1
**Phosphatase**	33^a^	33	14	3	2^a^	2	2	1
**Transcription regulator**	125	120	100^a^	19^b^	8	7	12^a^	9^b^
**Translation regulator**	14	17	3	0	1	1	0	0
**Transmembrane receptor**	41^a^	60^a^	13	1	3^a^	3^a^	2	0
**Transporter**	92	113	36	13^b^	6	6	4	6^b^

The unannotated probes have been removed from the calculations.

a Enrichments of the differentially expressed genes belonging to the category (p<0.001 and enrichment >1.5).

b More than 75% genes belonging to this specified category are upregulated (the categories with at least 10 genes).

### Bioinformatics analysis of cellular pathways affected by ZnO-1 exposure

Gene ontology (GO) analysis (**Table S6**) for the differentially expressed transcripts revealed 26 Biological Process terms to be enriched both in HMDM and Jurkat cells at 6 and 24 h timepoints representing the shared GO terms between these cell types independently of the timepoint of analysis (**Table 4**). Most of these terms represent genes regulating cell death or growth. Enrichment analysis also indicates that the ZnO-1 exposure leads to regulation of genes involved in the immune system development. In general, the ZnO-1 exposure causes activation of the immune response based on the observed trend of cytokine gene upregulation both in Jurkat and especially in HMDM cells (**Table S4**). Activation of unfolded protein response (UPR) suggests that 10 µg/ml ZnO-1 exposure leads to cell stress causing cells to activate their repair mechanisms. This finding may also shed light on the recent observation that ZnO-1 trigger apoptosis in Jurkat cells [7], as UPR is linked to apoptosis induction [35]. Moos *et al*. also noted activation of UPR by ZnO-EN exposed colon and skin cell lines [10]. To compare our results, we analyzed the expression of the reported ZnO-responsive genes among the samples used in our study. The clustering analysis shows that some of the reported genes separate the ZnO-1 treated cells from the controls also in our experimental setup. The analysis also shows that the transcriptional response to ZnO-EN is kinetic in general. Although there are cell type specific differences in the rate of transcriptional response (**Figure 4A**), the kinetic trends resemble each other in a variety of cell types. This is evident from a better overlap between the ZnO-EN responsive genes at 4 h reported by Moos *et al*. [10] and our Jurkat and HMDM samples treated with 10 µg/ml of ZnO-1 for 6 h than the other samples analyzed in the present study (**Figure S8, Table S7**). However, there were many genes, which did not respond in a similar way in these experiments. Comparison of the gene lists within the enriched GO-categories shows that ZnO-1 exposure regulates the same biological processes in HMDM and Jurkat cells, although the activity is often mediated via different set of genes. This emphasizes the importance of unbiased analysis methods, such as transcriptional profiling, and efficient usage of bioinformatics tools in characterization of EN responses.

**Table 4:** Common Biological Process terms enriched both in HMDM and Jurkat cells at 6 and 24 h timepoints.

	HMDM 6h		HMDM 24h		Jurkat 6h		Jurkat 24h	
Biological Process GO Term	Fold	p-value	Fold	p-value	Fold	p-value	Fold	p-value
GO:0010033∼response to organic substance	1.652	0.000	1.496	0.000	1.662	0.000	3.156	0.000
GO:0042981∼regulation of apoptosis	1.893	0.000	1.592	0.000	1.776	0.000	2.357	0.001
GO:0043067∼regulation of programmed cell death	1.875	0.000	1.589	0.000	1.758	0.000	2.334	0.001
GO:0010941∼regulation of cell death	1.868	0.000	1.583	0.000	1.780	0.000	2.325	0.001
GO:0016265∼death	1.936	0.000	1.322	0.004	1.542	0.003	2.380	0.001
GO:0008219∼cell death	1.932	0.000	1.317	0.004	1.553	0.003	2.397	0.001
GO:0051789∼response to protein stimulus	2.743	0.000	1.877	0.009	5.146	0.000	11.470	0.000
GO:0043066∼negative regulation of apoptosis	2.321	0.000	1.906	0.000	1.950	0.001	2.675	0.012
GO:0043069∼negative regulation of programmed cell death	2.289	0.000	1.879	0.000	1.923	0.001	2.637	0.013
GO:0060548∼negative regulation of cell death	2.283	0.000	1.874	0.000	1.917	0.001	2.630	0.014
GO:0043065∼positive regulation of apoptosis	1.886	0.000	1.525	0.001	1.983	0.000	2.426	0.015
GO:0043068∼positive regulation of programmed cell death	1.873	0.000	1.538	0.000	1.970	0.000	2.410	0.016
GO:0010942∼positive regulation of cell death	1.864	0.000	1.530	0.000	2.013	0.000	2.398	0.016
GO:0012501∼programmed cell death	2.113	0.000	1.318	0.009	1.715	0.001	2.196	0.011
GO:0040008∼regulation of growth	1.542	0.005	1.502	0.003	1.682	0.014	2.769	0.010
GO:0006915∼apoptosis	2.104	0.000	1.304	0.012	1.702	0.001	2.070	0.022
GO:0001558∼regulation of cell growth	1.702	0.008	1.604	0.010	2.129	0.005	3.407	0.017
GO:0006986∼response to unfolded protein	3.101	0.000	1.838	0.046	6.139	0.000	11.967	0.000
GO:0006916∼anti-apoptosis	2.506	0.000	1.714	0.002	1.791	0.034	3.224	0.021
GO:0008361∼regulation of cell size	1.484	0.049	1.560	0.013	2.005	0.009	3.666	0.006
GO:0002520∼immune system development	1.374	0.066	1.492	0.010	1.995	0.002	2.737	0.027
GO:0001775∼cell activation	1.464	0.025	1.803	0.000	1.939	0.003	2.327	0.079
GO:0048534∼hemopoietic or lymphoid organ development	1.411	0.053	1.545	0.006	1.941	0.005	2.542	0.057
GO:0009991∼response to extracellular stimulus	1.779	0.002	1.369	0.071	1.669	0.057	4.291	0.001
GO:0045859∼regulation of protein kinase activity	1.524	0.006	1.339	0.035	1.463	0.076	2.463	0.029
GO:0043549∼regulation of kinase activity	1.507	0.006	1.294	0.057	1.414	0.098	2.380	0.035

Fold: fold enrichment of the GO term based on comparison between the ratios of the differentially regulated genes that belong to a given GO-term and all genes measured that belong to this particular GO-term.

p-value: EASE score p-value indicating the statistical significance of the fold enrichment.

Table has been sorted based on the ascending sum of the p-values for each term.

The GO terms specifically enriched in either HMDM or Jurkat cells at both timepoints i.e. the most characteristic GO terms to each cell type independent of the length of ZnO-1 exposure, reveal clearly the functional difference of HMDM and Jurkat cells. The top Biological Process term in HMDM was “immune response” and in Jurkat cells “regulation of cell cycle” (**Table 5**). Then enriched Molecular Function terms regulated in HMDM or Jurkat cells were compared between the detection timepoints (**Table 6**). In HMDM, clear overrepresentation of the genes coding for ribosomal proteins and MHC class II molecules was found at 24 h. Most of the genes belonging to these categories were downregulated. Macrophages work at the interphase of innate and adaptive immunity, and the downregulation of the MHC class II molecules indicates that their capacity to present antigens may be compromised after ZnO-1 exposure. In Jurkat cells, the most significantly enriched Molecular Function is “aminoacyl-tRNA ligase activity” at 6 h. Aminoacyl tRNA synthetases are enzymes responsible for charging the tRNA with the amino acid. Interestingly, in addition to their conventional role, there is growing evidence linking these genes to many autoimmune diseases such as rheumatoid arthritis, and cancer [36].

**Table 5:** The top cell type-specific Biological Process terms enriched only in HMDM or Jurkat cells both at 6 and 24 h timepoints.

	6 h		24 h	
	Fold	p-value	Fold	p-value
**Jurkat**				
GO:0051726∼regulation of cell cycle	1.738	0.010	3.147	0.003
GO:0009612∼response to mechanical stimulus	4.097	0.001	6.744	0.021
GO:0051173∼positive regulation of nitrogen compound metabolic process	1.463	0.014	2.056	0.018
GO:0045935∼positive regulation of nucleobase, nucleoside, nucleotide and nucleic acid metabolic process	1.436	0.022	2.122	0.014
GO:0045941∼positive regulation of transcription	1.467	0.021	2.180	0.016
GO:0010557∼positive regulation of macromolecule biosynthetic process	1.443	0.018	2.027	0.020
GO:0031328∼positive regulation of cellular biosynthetic process	1.442	0.015	1.932	0.028
GO:0009891∼positive regulation of biosynthetic process	1.424	0.018	1.907	0.031
GO:0010628∼positive regulation of gene expression	1.424	0.031	2.116	0.019
GO:0042326∼negative regulation of phosphorylation	3.059	0.045	8.392	0.012
**HMDM**				
GO:0006955∼immune response	1.715	0.000	2.174	0.000
GO:0009611∼response to wounding	1.781	0.000	2.000	0.000
GO:0050867∼positive regulation of cell activation	2.755	0.000	2.533	0.000
GO:0002696∼positive regulation of leukocyte activation	2.769	0.000	2.557	0.000
GO:0008285∼negative regulation of cell proliferation	1.863	0.000	1.724	0.000
GO:0050865∼regulation of cell activation	2.236	0.000	2.123	0.000
GO:0006954∼inflammatory response	1.835	0.000	2.322	0.000
GO:0051251∼positive regulation of lymphocyte activation	2.900	0.000	2.484	0.000
GO:0042330∼taxis	2.477	0.000	2.097	0.000
GO:0006935∼chemotaxis	2.477	0.000	2.097	0.000

Fold: fold enrichment of the GO term based on comparison between the ratios of the differentially regulated genes that belong to a given GO-term and all genes measured that belong to this particular GO-term.

p-value: EASE score p-value indicating the statistical significance of the fold enrichment.

Table has been sorted based on the ascending sum of the p-values for each term.

**Table 6:** The most enriched timepoint-specific Molecular Function terms in HMDM or Jurkat cells either at 6 or 24 h timepoints.

	Fold	p-value
**HMDM 6 h**		
GO:0016866∼intramolecular transferase activity	4.88	0.000
GO:0019899∼enzyme binding	1.50	0.001
GO:0017076∼purine nucleotide binding	1.22	0.001
GO:0004721∼phosphoprotein phosphatase activity	1.96	0.002
GO:0005096∼GTPase activator activity	1.75	0.003
GO:0032553∼ribonucleotide binding	1.20	0.004
GO:0032555∼purine ribonucleotide binding	1.20	0.004
GO:0000166∼nucleotide binding	1.17	0.005
GO:0003711∼transcription elongation regulator activity	4.07	0.006
GO:0016791∼phosphatase activity	1.64	0.006
**HMDM 24 h**		
GO:0003735∼structural constituent of ribosome	2.590	0.000
GO:0032395∼MHC class II receptor activity	8.422	0.000
GO:0046915∼transition metal ion transmembrane transporter activity	3.743	0.001
GO:0000287∼magnesium ion binding	1.457	0.002
GO:0046983∼protein dimerization activity	1.393	0.003
GO:0008092∼cytoskeletal protein binding	1.400	0.004
GO:0015082∼di-, tri-valent inorganic cation transmembrane transporter activity	2.850	0.004
GO:0015149∼hexose transmembrane transporter activity	3.930	0.006
GO:0042802∼identical protein binding	1.319	0.007
GO:0015145∼monosaccharide transmembrane transporter activity	3.723	0.008
**Jurkat 6 h**		
GO:0004812∼aminoacyl-tRNA ligase activity	5.977	0.000
GO:0016875∼ligase activity, forming carbon-oxygen bonds	5.977	0.000
GO:0016876∼ligase activity, forming aminoacyl-tRNA and related compounds	5.977	0.000
GO:0003723∼RNA binding	1.727	0.000
GO:0003677∼DNA binding	1.335	0.000
GO:0051087∼chaperone binding	6.973	0.000
GO:0060590∼ATPase regulator activity	12.729	0.000
GO:0000049∼tRNA binding	6.874	0.001
GO:0015175∼neutral amino acid transmembrane transporter activity	6.546	0.002
GO:0070035∼purine NTP-dependent helicase activity	2.835	0.003
Jurkat 24 h		
GO:0004860∼protein kinase inhibitor activity	12.965	0.001
GO:0019210∼kinase inhibitor activity	12.615	0.001
GO:0019207∼kinase regulator activity	6.088	0.003
GO:0019887∼protein kinase regulator activity	5.834	0.010
GO:0004857∼enzyme inhibitor activity	2.861	0.021
GO:0004672∼protein kinase activity	2.023	0.026
GO:0004674∼protein serine/threonine kinase activity	2.196	0.038
GO:0047115∼trans-1,2-dihydrobenzene-1,2-diol dehydrogenase activity	46.674	0.042
GO:0032403∼protein complex binding	2.858	0.058
GO:0001948∼glycoprotein binding	7.569	0.059

Fold: fold enrichment of the GO term based on comparison between the ratios of the differentially regulated genes that belong to a given GO-term and all genes measured that belong to this particular GO-term.

p-value: EASE score p-value indicating the statistical significance of the fold enrichment.

Table has been sorted based on the ascending sum of the p-values for each term.

### Upstream regulators of ZnO-1-derived gene expression

In order to find out transcriptional mediators for ZnO-1 derived toxicity, we used Ingenuity Pathway Analysis software to identify the transcription factors reported to regulate the genes differentially expressed in our data. Transcription factor having the most significant overlap of the target molecules in the dataset in HMDM and Jurkat cells when 6 and 24 h data were combined was glucocorticoid receptor, NR3C1. Altogether, it had 106 and 45 target genes in HMDM and Jurkat cells, respectively (**Table S4**). 19 of these NR3C1 target genes were regulated in both cell types. Glucocorticoids are known to mediate several signaling pathways leading to apoptosis of T lymphocytes [37]. Steroid hormones are the most potent activators of glucocorticoid receptor, but it is also evident that cell stress can lead to ligand-independent activation of glucocorticoid receptor [38]. Connection between glucocorticoid receptor and zinc-induced gene regulation has been previously shown in pancreatic cells in which MTF-1 and glucocorticoid receptor regulate zinc transporter *Slc30a2* expression [39]. As zinc, on the other hand, has been shown to inhibit glucocorticoid receptor ligand binding [40], and either promote or block apoptosis [41], there most likely is a complex interplay between zinc and glucocorticoid signaling regulating cell death *in vivo*. Our results suggest that this connection should be carefully dissected.

Recently, Hanagata *et al*. [42] proposed that A549 lung epithelial cells avoid cell death caused by Cu ions released from CuO-ENs by arresting cell cycle. Our data show that the most highly downregulated gene in Jurkat cells after 6 h of exposure to ZnO-1 was *MYC*. MYC is a potent driver of cell proliferation and growth, and it regulates apoptosis [43]. As at least 15% of genes are estimated to be regulated by this transcription factor [44], it was of interest to analyze whether there were known MYC targets regulated in our data. We found seven MYC targets, which were regulated both in HMDM and in Jurkat cells (**Table S4**). Most important of these, in context of cell cycle regulation, was *CDKN1A*, which mediates cell cycle arrest to G1 phase. The expression of *CDKN1A* was upregulated in HMDM and Jurkat cells at 6 and 24 h timepoint. Regulation of *MYC* was not detected in HMDM or Jurkat cell samples at 24 h timepoint, indicating that ZnO-1 cause cell and time dependent MYC response. In contrast to the conclusions drawn by Hanagata *et al*. [42], our transcriptomics data showed that several p53 target genes were regulated following ZnO-1 exposure (**Table S4**). There were 101 and 49 reported p53 target genes regulated in HMDM and in Jurkat cells, respectively. Out of these 21 were common between the cell types. Regulation of p53 itself cannot be seen, but phosphorylation and decreased protein turnover are the most critical mechanisms for p53 activation [45]. p53 activation leads to cell cycle arrest and apoptosis if a cell cannot repair detected cellular damages. In the *in vitro* culturing model used in this study, part of the p53 effects can be also indirect, because zinc regulates p53 folding [46].

### Validation of the results

ZnO-1 had an effect only on 12 genes in MDDC samples based on the genome-wide gene expression profiling. To confirm this unexpected deviation from the other cell types analyzed, we hybridized the MDDC samples treated with 10 µg/ml of ZnO-1 for 24 h to Affymetrix GeneChip Human Genome U219 array plates (**Figure S9**). In addition, we processed four new independent MDDC treatment-control sample pairs for the analysis (**Table S3**). Affymetrix U219 array is the most updated genome-wide microarray available and due to the different probe design can be used to validate Illumina results. The low transcriptional response to ZnO-1 exposure is evident even with a larger MDDC sample set (n = 7) (**Figure S10, Table S8**), the finding being in-line with the reported unaltered phenotype of ZnO-1 exposed MDDC [23]. Importantly again, differences between the individuals override the common gene expression pattern (**Figure S10**). This result highlights the challenge of using primary cells from different donors over the transformed cell lines. Although *in vitro* data gathered with primary cells reflects better *in vivo* situation of nanoparticle exposure, variation between individuals might limit ability to observe generic particle-specific effects in small study groups. On the other hand, because there were donor-specific ZnO-1 responses, individual testing is needed (**Figure S11**). In a broader context, variation between replicate experiments may also arise for example from usage of different EN batches, cell culture products and cell line passages. These kinds of parameters should be controlled as much as possible to avoid non-biological variation in data. On average, the Affymetrix experiment validated the differential expression of 82% of the probes (range 60–100%) with the fold change >1.5 criterion among all cell types studied (**Table S4**).

**Figure 6:**
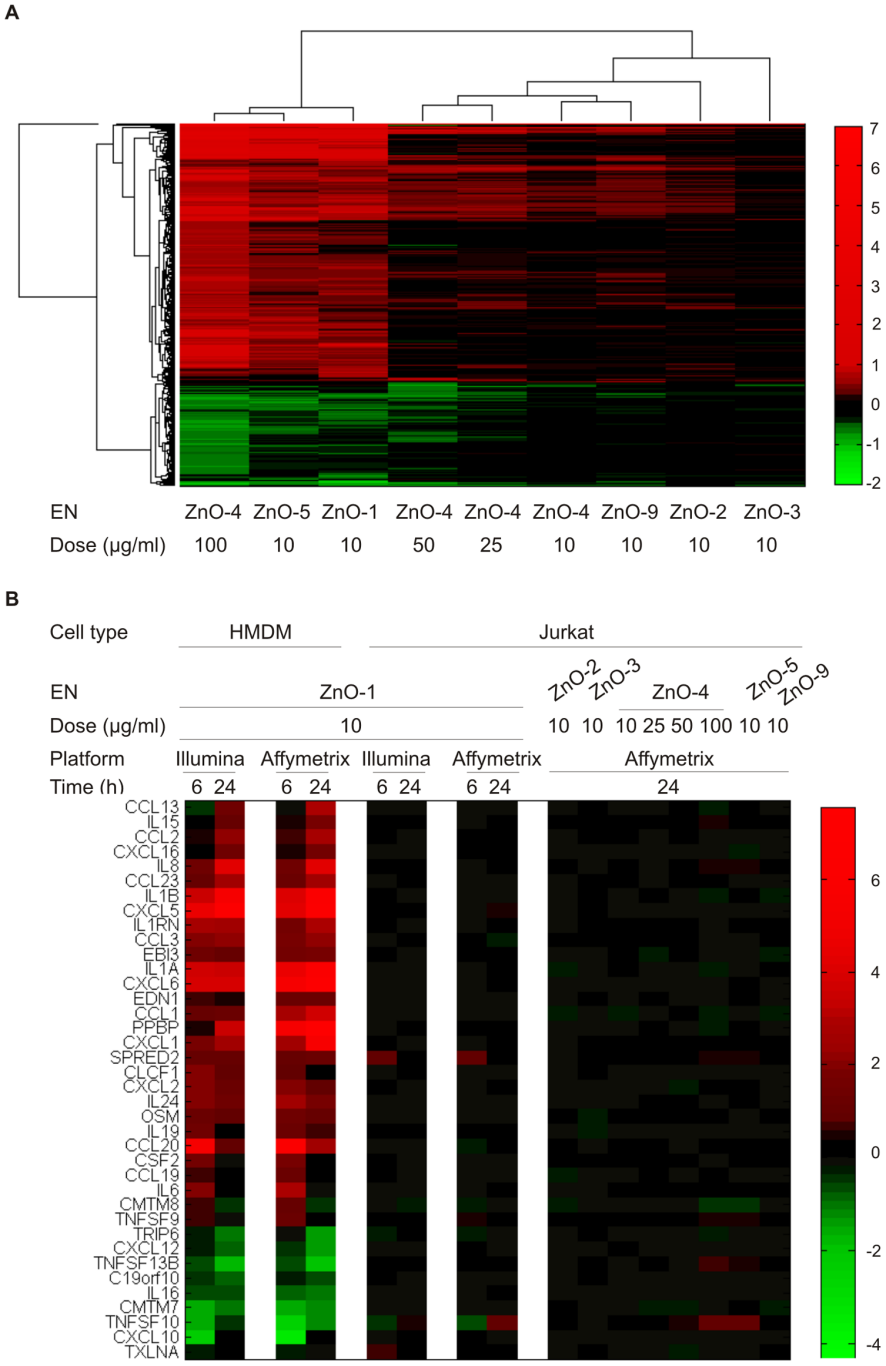
Comparison of transcriptional response caused by different ZnO nanoparticles. A) The response of Jurkat cells to differentially modified ZnO nanoparticles was analyzed with in three replicate experiments. The figure shows the fold changes of the differentially expressed genes throughout the treatments clustered with correlation distance and complete linkage. The rows present the genes detected as differentially expressed at least in one of the comparisons. The characteristics of the ZnO nanoparticles are listed in Table 1, Table S1 and Table S2. B) Expression of the genes annotated as cytokines in the Ingenuity Knowledge Base and detected as differentially expressed in response to ZnO-1 EN exposure in HMDM or Jurkat cells. The expression of the genes is shown with the Illumina and the Affymetrix platforms. The Illumina data represents the probe having the biggest difference between the treated and the control samples. Only the genes measured with both of the platforms are shown. In both of the figures the columns represent the logarithmic fold change value of the limma analysis when the EN-treated samples were compared against the non-treated control cells.

To further elucidate cellular responses to ZnO-ENs, we compared the panel of surface-modified ZnO-ENs to determine whether the physico-chemical characteristics of the particles affect the global transcriptional response. Jurkat cells were exposed altogether to five different ZnO-ENs (ZnO-2, ZnO-3, ZnO-4, ZnO-5 and ZnO-9) (**Table S3**). The selected ZnO-ENs were of different size, morphology and surface chemistry resulting different aggregation tendency (**Table 1** **and** **2** **, Figure S2, Table S2**). As leaching rate and efficiency are dependent on the composition of culturing medium and surface chemistry of the modified nanoparticles used, instead of direct comparison of the effects of different zinc ion concentration on the gene expression profiles we employed ZnO-4 dose-response to extrapolate the effects of different concentrations of released zinc. Jurkat cells were exposed to ZnO-4 with four different subtoxic concentrations [7] and the samples were collected at 24 h timepoint. Hierarchical clustering of the results along with the ZnO-1 data revealed that, of the modified particles studied, ZnO-5 caused the biggest transcriptional deviation compared to the control cells (**Figure 6A**). Transcriptional response of ZnO-1 (10 µg/ml), ZnO-5 (10 µg/ml) and ZnO-4 (100 µg/ml) separate from the rest of the samples; both the magnitude of the changes and the number of differentially expressed genes is the highest after these treatments (**Figure S12, Table S8**). This is in line with the cell viability data. Clustering of the data also illustrates that the genes regulated by each ZnO-EN are largely the same. For example, when the expression of the cytokines is examined over the datasets, it is evident that the immunological response to different ZnO-ENs is comparable (**Figure 6B**). Analysis also validates the cell-type specificity of the response. In summary, the analysis confirms that the exposure with different ZnO-ENs causes highly related gene expression profile, which is comparable to the amount of EN used in the exposure, and reflects the toxicity of the ENs.

Several previous studies with different metal oxide ENs have indicated that toxicity of the ENs is derived from leached metal ions [7]–[9], [47], [48]. On contrary, there has been a report showing that ZnO-EN has to be in touch with colon cells for causing toxicity [10]. A follow-up study from the same research group shows that ZnCl_2_ and zinc deliberated from ZnO-EN cause a distinguishable gene expression pattern compared to direct ZnO-EN exposure [10]. It remains to be studied whether ZnO-ENs has some contact dependent effects also in HMDM, MDDC and Jurkat cells. However, the ion chelation analysis and comparison to ZnCl_2_ treatment have shown that leaching of the ions is clearly the main mechanism for ZnO-1 derived cell death in Jurkat cells [7]. In the experimental set up used in our study, we have not detected ZnO-1 inside HMDM, MDDC or Jurkat cells [7], [23], which is in contrast to the conclusions done by Moos *et al*. with RKO and SK Mel-28 cells [10]. Recently, Gilbert *et al*. applied several complementary imaging techniques to show that in human bronchial epithelial BEAS-2B cells ZnO-ENs are taken up by the cells and dissolution occurs rapidly inside the cells [48].

Transcriptional response to excess or deprivation of zinc has been directly studied with human THP-1 cell line [28]. When the expression of the reported zinc responsive genes are analyzed in our samples, distinct ZnO-EN specific clusters can be found within HMDM and Jurkat ZnO-1 treated cells (**Figure S13, Table S9**). In Jurkat cells the 6 h samples treated with 10 µg/ml of ZnO-1 are in a separate cluster from the other samples. In HMDM both the 6 h and 24 h 10 µg/ml ZnO-1 treated cells form a separate expression clusters. As Cousins *et al*. [28] analyzed the cells after 4 h of zinc-treatments, the correlation between the findings reported in their study with both 6 h ZnO-1 treated HMDM and Jurkat cells highlights again the kinetic fashion of transcriptional changes in response to variations in zinc levels. Overall, the comparison of the responses of different ZnO-ENs and overlap of the data with the reported zinc target genes support the determining role of Zn^2+^ ions to ZnO-EN-derived gene expression.

## Conclusions

The data reported herein reveal that the same nanoparticles at the identical dose and exposure time may elicit markedly different responses at the transcriptional level in different cell types of immune origin, thus underscoring the need for unbiased profiling of target genes and pathways affected by different ENs. Due to the variable responses of different cell types, it might be challenging, or even impossible, to construct a compact panel of readouts, which could be used to assess all the aspects of the responses of certain EN in all cell types. Notwithstanding, our data also demonstrate that the upregulation of metallothionein genes in response to ZnO-1 exposure is a common gene “signature” that is shared among MDDC, HMDM and Jurkat cells. We and others have shown that particle dissolution drives toxicity of ZnO-ENs [7], [8]. The results presented here give additional proof by using global transcriptomics that the cellular response to ZnO-ENs is due to leached Zn^2+^ ions. As such, our study describes an approach for the assessment of EN derived responses starting from toxicological cell viability analysis to genome-wide profiling. Such global toxicogenomic studies, in combination with thorough bioinformatics assessment of the data, form a basis for identification of signaling pathways affected by ENs, and may thus further increase our understanding of nano-bio-interactions. This information is a prerequisite for the safe use of nanomaterials.

## Materials and Methods

### Ethics Statement

Primary human blood cells were isolated from buffy coats obtained from healthy adult blood donors at the Blood Transfusion Center, Karolinska University Hospital, Stockholm, Sweden. The donors are approved and covered by insurance according to the regulations at the University Hospital. These buffy coats contain white blood cells and are a waste product after the red blood cells have been utilized for blood transfusions. The identity of the blood donors is unknown to the scientists performing the experiments. In addition, genetic (i.e. transcriptomics) data are stored without any personal identifiers and the data cannot be traced back to the individual blood donors. Prior to the present study, advice was sought from the Ethical Committee for Human Studies in Stockholm, and a statement was issued that there are no objections to studies of nanomaterials on cells derived from human buffy coats, since the data cannot be traced back to the individual blood donors; hence, no specific ethical permit is required (see #2006/900-31/3, decision 2006/3:8).

### Nanoparticles

ZnO-1 was commercially available from IBU-tec advanced materials AG and was compared to ZnO-2 (mandelic acid modified), ZnO-3 (mercaptopropyl-trimethoxysilane modified), ZnO-4 (methoxyl modified), ZnO-5 (diethylene glycol modified), ZnO-6 (mandelic acid modified), ZnO-7 (gluconic acid modified), ZnO-8 (citric acid modified) and ZnO-9 (folic acid modified). Commercial TiO_2_ was from Evonik Degussa (Aeroxide® TiO2 p25). A detailed description of the production and characterization of the different nanomaterials is presented in Methods S1 and in our previous publication [7]. Nanoparticles were dispersed by sonication in ultra-pure water to a stock solution of 1 mg/ml. Due to the potential dissolution of ZnO nanoparticles, stock suspensions were prepared just prior to the experiments and immediately added to the cell cultures at the required experimental dilutions. The nanoparticles were controlled for lipopolysaccharide (LPS) contamination [49] by using the chromogenic LAL test method (Limulus Amebocyte Lysate Endochrome, Charles River Endosafe) according to the manufacturer's instructions.

### Analysis of ZnO-EN dissolution

The particles were introduced into the cell culturing medium in a concentration of 10 µg/ml and ultrasonicated for 5 min (70 W, 20 kHz, Ultrasonic Homogenizer Bandelin Sonopuls UV 70). The mixtures were kept in an oil bath at 37°C in capped centrifuge tubes with constant magnetic stirring (100 rpm) for 30 min, 6 h or 24 h. For the removal of the solid particles, the tubes were centrifuged altogether three times at 11 000 rpm for 10 minutes with Eppendorf-Centrifuge 5804. After the separation, the supernatant was carefully transferred into another tube for two consecutive centrifugations to ascertain the purity of the soluble fraction. Measurements were done with iCE 3500 Atomic Absorption Spectrometer (Thermo Fisher Scientific). The presented data is an average of three independent measurements.

### Jurkat cell culture and generation of HMDM and MDDC

Jurkat A3 (ATCC, CRL-2570) cells were cultured in Roswell Park Memorial Institute medium (RPMI) 1640 culture medium (Sigma) supplemented with 10% fetal calf serum, 2 mM L-glutamine (Gibco) and 1% penicillin-streptomycin-neomycin (PSN) (Gibco) as indicated in Buerki-Thurnherr *et al*. [7] The detailed protocol for generation of the HMDM and MDDC and the controlling of the phenotypes are described in the publication by Kunzmann *et al*. [50] Shortly, peripheral blood mononuclear cells (PBMC) were prepared from buffy coats obtained from healthy blood donors (Karolinska University Hospital, Stockholm, Sweden) by density gradient centrifugation and positively selected for CD14 expression (CD14 MicroBeads, human (Miltenyi Biotec, Bergisch Gladbach, Germany)). To obtain HMDM, CD14+ monocytes were cultured in supplemented RPMI 1640 medium (Sigma Aldrich) with 50 ng/ml recombinant M-CSF (Novakemi, Handen, Sweden) for three days. To obtain immature MDDC, CD14+ monocytes were cultured at a density of 4×10^5^ cells/ml at 37°C in a humidified atmosphere containing 6% CO_2_ in supplemented RPMI 1640 medium (Sigma Aldrich) with IL-4 (800 IU/ml) and GM-CSF (550 IU/ml) (Biosource International, Camarillo, CA). After three days, half of the culture medium was exchanged with fresh medium containing the cytokines. After 6 days, the cells were collected for nanoparticle exposure. The MDDC and HMDM samples used in this study were derived from the cells of different donors.

### Exposure of HMDM, MDDC and Jurkat cells to nanoparticles

HMDM and Jurkat cells were seeded in 24-well plates at a density of 1×10^6^ cells/well and 1.5×10^5^ cells/well respectively in a final volume of 1 ml and treated with the indicated concentrations of nanoparticles for the indicated times [7]. Immature MDDC were seeded in 6-well plates at a density of 4×10^5^ cells/ml in a final volume of 3 ml [23]. Nanoparticle exposures were done in the culturing mediums without any cytokines. Cell viability was assessed as indicated in Methods S1 or as previously described [7], [23].

### Microarray sample preparation

Cells were collected to RNAlater buffer (Ambion) and total RNA isolated with RNAqueous Small Scale Phenol-Free Total RNA Isolation Kit (Ambion) according to manufacturer's protocol. For Illumina microarray analysis 300 ng of RNA was amplified with Illumina RNA TotalPrep Amplification kit (Ambion). *In vitro* transcription, during which cRNA was biotinylated, was carried out for 16 h. Both before and after the amplifications the RNA/cRNA concentrations where checked with Nanodrop ND-1000, and integrity and amplification profile controlled with BioRad's Experion Automated Electrophoresis System (Bio-Rad). 0.75 µg labeled cRNA was hybridized to Illumina's Sentrix HumanHT-12 Expression BeadChips, version 3 (cat no BD-103-0603) at 58°C for 18 h according to Illumina Whole-Genome Gene Expression Direct Hybridization protocol, revision A. The order of the samples was randomized to avoid sample location biases [51]. Hybridization was detected with 1 µg/ml Cy3-Streptavidin (GE Healthcare). The arrays were scanned with Illumina BeadArray Reader, BeadScan software version 3.5 and the numerical results extracted with GenomeStudio 2009.2 without any normalization.

For Affymetrix analysis 250 ng of total RNA was processed with GeneChip 3′ IVT Express Kit (Part no. 901229) and hybridized to GeneChip Human Genome U219 array plate with specific protocols for using the GeneTitan Hybridization, Wash and Stain Kit for 3′ IVT Array Plates (P/N 901530). GeneTitan Instrument was used to hybridize, wash, stain and scan the arrays. Affymetrix GeneChip Command Console 3.1 was used to control the process and to summarize probe cell intensity data. Hybridization quality was checked with Affymetrix GeneChip Command Console and Expression ConsoleTM 1.1s.

### Microarray analysis

The data of the samples measured with the Illumina's Sentrix HumanHT-12 Expression BeadChips were analyzed using R and the Bioconductor Lumi package [52]. The raw data values were pre-processed with the Variance Stabilizing Transform (VST) of Illumina data [53]. Further, the values were between-chip normalized with the quantile normalization method [54]. Samples analyzed with Affymetrix platform were preprocessed using the Robust Multi-array Average (RMA) algorithm using the R package affy [55]. The probe values were linked directly into the ENSEMBL genes with Brainarray CDF-files Version 14 [56]. To ensure the independent analyses of EN-specific gene expression changes in MDDC, HMDM, and Jurkat cells, the different cell types were preprocessed separately. In contrast, to compare the untreated 6 h samples, all the samples from each cell type were preprocessed together from the raw values.

Differentially expressed genes were detected with the limma package [22] of R using empirical Bayes moderated t-test and the p-values were adjusted with the FDR method [57]. The limma analyses were performed as paired analysis, since all the samples were naturally paired with their non-treated replicate. Thus, the sib-pair effect was taken into account in the linear model. All the genes with the adjusted p-value (FDR) less than 0.05 and absolute fold change at least 1.5 between the compared groups were assigned as differentially expressed. Affymetrix Human Genome U219 array plate results were used to validate the key findings of the microarray analysis done with Illumina arrays. Thus, the differential expression of the genes highlighted in the text was validated either with RT-PCR or hybridization with Affymetrix microarrays. Ingenuity Pathway Analysis software (www.ingenuity.com) was used in data annotation and identification of transcription regulators. The data produced in this study is available from the Gene Expression Omnibus database (GSE39444).

### RT-PCR

Isolated RNA was treated with DNase I Amplification Grade (Invitrogen). cDNA was synthesized with Transcriptor First Strand cDNA Synthesis Kit (Roche). qPCR was performed using Universal ProbeLibrary probes (Roche Applied Science) and custom ordered oligos designed with Universal ProbeLibrary Assay Design Centre or with FAM (reporter), TAMRA (quencher) double labeled probe in 10 µl reaction volume. The primers and probes are listed in the **Table S10**. Reaction mix used was ABsolute QPCR ROX Mix (Thermo Scientific) and amplification was monitored with Applied Biosystems 7900HT Fast Real-Time PCR System (15 min enzyme activation and 40 cycles of 15 s 95°C, 1 min 60°C). The fold changes of the transcripts were calculated by using the equations: ΔCt = (CtGene−CtEF1α), Δ(ΔCt) = (ΔCt(EN)−ΔCt(Ctr)) and fold change = 2ˆ|Δ(ΔCt)|. In the equations, the Ct is a cycle threshold value at which the RT-PCR signal exceeds the detection threshold. The Ct-values of the transcripts studied were normalized against the signals acquired with *EF1α* [58]. If the expression of a gene was undetectable in the untreated control samples, the Ct-value 35 was given to it before fold change calculations. Statistical significance of the findings was tested with t-test and the p-value 0.05 was considered as the limit of significance.

## Supporting Information

Figure S1 **Size distribution charts of the ENs.** Transmission electron microscopy (TEM) was used to investigate the size of the ENs. Hundred particles were counted for each sample. Size distribution charts presented show the particle size frequencies, an average diameter and a calculated statistical standard deviation acquired with non-linear fitting of the data for each sample.
(PDF)

Figure S2 **TEM analysis of ZnO nanoparticles.** TEM micrographs of A) ZnO-1, B) ZnO-2, C) ZnO-3, D) ZnO-4, E) ZnO-5, F) ZnO-6, G) ZnO-7, H) ZnO-8, I) ZnO-9 and J) TiO_2_.
(PDF)

Figure S3 **XRD pattern of ZnO nanoparticles.** XRD spectra for ZnO-5, ZnO-6, ZnO-7, ZnO-8 and ZnO-9. They all reveal the typical peaks of hexagonal wurtzite type crystal structure. XRD results for ZnO-1, ZnO-2, ZnO-3, ZnO-4 and TiO_2_ have been presented in Buerki-Thurnherr *et al*. [7].
(PDF)

Figure S4 **Schematic presentation of the experimental set-up for Illumina microarray hybridizations.** HMDM samples are used as an example in the figure. Similar experiments were performed with MDDC and Jurkat cells. The red dotted arrows indicate the basic comparisons done for the microarray data. All experiments were repeated three times with independent cell cultures and analyzed with Illumina Sentrix HumanHT-12 Expression BeadChips. The samples marked with blue boxes were hybridized also to Affymetrix GeneChip Human Genome U219 array plates to validate the observations done with the Illumina arrays (**Figure S9**). Cell images from Biomedical PowerPoint Toolkit Suite (www.motifolio.com) were utilized in the figure preparation.
(PDF)

Figure S5 **MTT analysis of HMDM.** Toxicity of ZnO-1 and TiO_2_ ENs were assessed with MTT assay. ZnO-1 caused dose-dependent loss of cell viability whereas TiO_2_ was inert. The figure shows an average and the standard deviation of three independent cultures. Similar trends in toxicity were obtained using MDDC [23] and the Jurkat cells [7]. The cell viability of HMDM, MDDC and Jurkat cells with 10 µg/ml of ZnO-1 at 24 h timepoint was around 55%, 70%, and 70%, respectively.
(PDF)

Figure S6 **ZnO-1 and TiO_2_ induced gene expression in HMDM, MDDC and Jurkat cells.** The number of genes having on average at least 1.5-fold difference in expression between the nanoparticle exposed and the untreated control cells in the whole-genome microarray analysis with Illumina Sentrix HumanHT-12 Expression BeadChips. The cell type, timepoint and the doses are indicated in the figure. The red bars represent the genes, which were upregulated after nanoparticle treatment and the green the downregulated ones. Three replicates of each treatment were used in the analysis.
(PDF)

Figure S7 **Expression of the metallothioneins validated with RT-PCR in response to ZnO-1 and TiO_2_ nanoparticles.** Figure shows the normalized log2-transformed average expression levels in untreated control MDDC, HMDM and Jurkat cells and the expression after exposure of the cells with 10 µg/ml of ZnO-1 or 10 µg/ml of TiO_2_ at 6 and 24 h of culture. The data is from three replicate experiments.
(PDF)

Figure S8 **Hierarchical clustering of the expression of the ZnO responsive genes reported by Moos** ***et al*** **.** [10] among A) HMDM samples and B) Jurkat samples in Illumina Sentrix HumanHT-12 Expression BeadChip data after exposing the cells with 1 µg/ml or 10 µg/ml of ZnO-1 or TiO_2_ for 6 or 24 h in three replicate experiments (see **Figure S4** and **Table S3**). The subclusters discussed in the main text are highlighted. The gene expression data for the differentially expressed genes is shown in **Table S4.**
(PDF)

Figure S9 **Schematic presentation of the experimental set-up for Affymetrix GeneChip Human Genome U219 array hybridizations.** MDDC samples from four donors treated for 24 h with 10 µg/ml of ZnO-1 or cultured without exposure were hybridized to Affymetrix arrays. At the same time ZnO-1 (10 µg/ml) or untreated control cells cultured for 6 or 24 h from three replicate experiments of MDDC, HMDM and Jurkat cells previously analyzed with Illumina arrays were processed for array validation (**Figure S4**, **Table S3**). In addition, samples from Jurkat cells treated with ZnO-2, ZnO-3, ZnO-5 or ZnO-9 (10 µg/ml, 24 h), or ZnO-4 (10, 25, 50 or 100 µg/ml, 24h) were analyzed. The red dotted arrows indicate the basic comparisons done for the microarray data. Cell images from Biomedical PowerPoint Toolkit Suite (www.motifolio.com) were utilized in the figure preparation.
(PDF)

Figure S10 **ZnO-1 response in MDDCs.** A) Hierarchical clustering of the ZnO-1 (10 µg/ml) treated, or the untreated control MDDC samples using correlation distance and complete linkage in clustering. Seven different donors were used in the analysis. The gene expressions were measured with Affymetrix GeneChip Human Genome U219 array plates. The samples from the donors 1–3 are the same samples hybridized to Illumina Sentrix HumanHT-12 v3 Expression BeadChips, thus being platform independent technical replicates. The name of the sample is formed by combining the information about cell type, number of replicate in brackets, treatment and timepoint. B) The overlap of the detected statistically (FDR≤0.05 and absolute FC≥1.5) differentially expressed genes between the ZnO-1 treated cells and the untreated control cells at the indicated timepoints measured either with Affymetrix GeneChip Human Genome U219 array plates or Illumina Sentrix HumanHT-12 v3 Expression BeadChips. The figure was drawn with VENNY [59]. C) Hierarchical clustering results of all the samples analyzed with Illumina microarrays; HMDM, MDDC or Jurkat cells exposed with 1µg/ml or 10µg/ml of ZnO-1 or TiO_2_ for 6 h or 24 h, (see **Figure S4** and **Table S3**) by using correlation distance with complete linkage.
(PDF)

Figure S11 **Donor or replicate culture -specific responses to ZnO-1.** Venn diagrams of the differentially expressed genes in MDDC, HMDM and Jurkat cells (FC>1.5 or <−1.5) in each donor or replicate experiment at 6 h and 24 h timepoints after exposure to 10 µg/ml of ZnO-1 compared to the corresponding untreated control cells analyzed with Illumina Sentrix HumanHT-12 Expression BeadChips.
(PDF)

Figure S12 **Hierarchical clustering of the samples analyzed with Affymetrix GeneChip Human Genome U219 array plates.** The data is from three replicate experiments of Jurkat cells exposed with 10 µg/ml of ZnO-2, ZnO-3, ZnO-5 or ZnO-9 for 24 h; or 10 µg/ml, 25 µg/ml, 50 µg/ml or 100 µg/ml of ZnO-4 for 24 h; HMDM, MDDC or Jurkat cells exposed with 10 µg/ml of ZnO-1 for 6 h and 24 h, and 4 replicates experiments of MDDC exposed with 10 µg/ml of ZnO-1 for 24 h (see **Figure S4**, **Figure S9** and **Table S3**). The clustering was done by using correlation distance with complete linkage.
(PDF)

Figure S13 **Hierarchical clustering of the expression of the zinc responsive genes reported by Cousins** ***et al*** **.** [28] among A) HMDM samples and B) Jurkat samples in Illumina Sentrix HumanHT-12 Expression BeadChip data after exposing the cells with 1 µg/ml or 10 µg/ml of ZnO-1 or TiO_2_ for 6 or 24 h in three replicate experiments (see **Figure S4** and **Table S3**). The subclusters separating different treatments are highlighted. The gene expression data for the differentially expressed genes is shown in **Table S9.**
(PDF)

Table S1 **XRD, TGA and FT-IR analysis of the ZnO ENs.**
(XLSX)

Table S2 **Physicochemical characterization of the nanoparticles.**
(XLSX)

Table S3 **The samples used in the study.**
(XLSX)

Table S4 **The differentially expressed genes in the Illumina study.**
(XLSX)

Table S5 **Normalized expression of SLC30A and SLC39A family members.**
(XLSX)

Table S6 **GO term enrichment.**
(XLS)

Table S7 **Overlap of our findings with Moos** ***et al.*** **2011 dataset.**
(XLSX)

Table S8 **The differentially expressed genes in the Affymetrix study.**
(XLSX)

Table S9 **Overlap of our findings with Cousins** ***et al.*** **2003 dataset.**
(XLS)

Table S10 **RT-PCR reagents.**
(XLSX)

Methods S1 **Supplementary methods containing nanoparticle production and characterization, and cell viability analysis methods.**
(DOCX)
