# Supplementary material for: Gene Expression Profiling of Immune-Competent Human Cells Exposed to Engineered Zinc Oxide or Titanium Dioxide Nanoparticles
Source: PLoS One. 2013 Jul 22;8(7):e68415. doi: 10.1371/journal.pone.0068415 (PMC3718780; doi:10.1371/journal.pone.0068415)
Supplement: Figure S1 — Size distribution charts of the ENs. Transmission electron microscopy (TEM) was used to investigate the size of the ENs. Hundred particles were counted for each sample. Size distribution charts presented show the particle size frequencies, an average diameter and a calculated statistical standard deviation acquired with non-linear fitting of the data for each sample. (PDF) [file pone.0068415.s001.pdf]

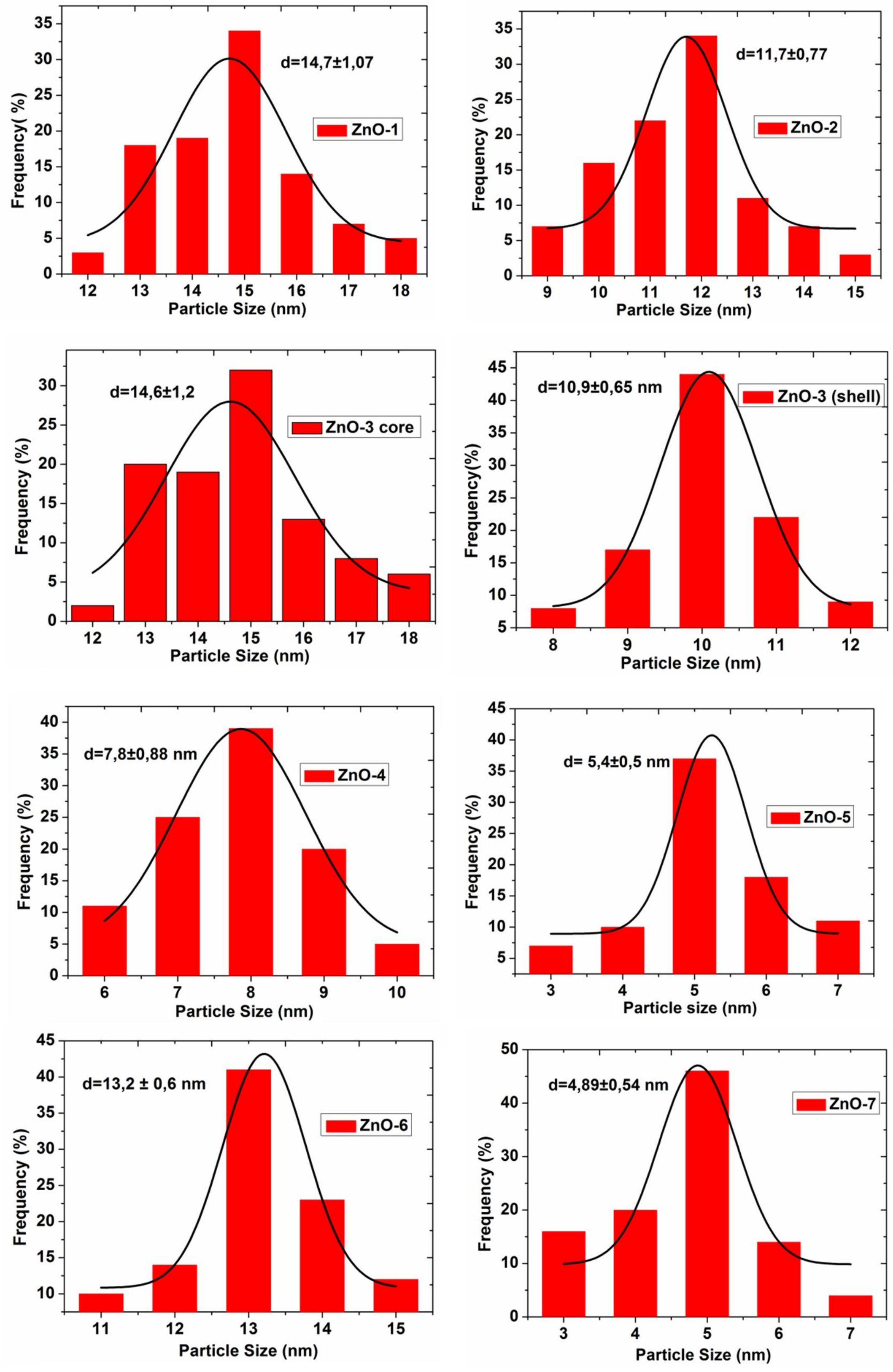

d=14,7±1,07
ZnO-1
Frequency(%)
Particle Size (nm)
d=11,7±0,77
ZnO-2
Frequency (%)
Particle Size (nm)
d=14,6±1,2
ZnO-3 core
Frequency (%)
Particle Size (nm)
d=10,9±0,65 nm
ZnO-3 (shell)
Frequency(%)
Particle Size (nm)
d=7,8±0,88 nm
ZnO-4
Frequency (%)
Particle Size (nm)
d= 5,4±0,5 nm
ZnO-5
Frequency (%)
Particle size (nm)
d=13,2 ± 0,6 nm
ZnO-6
Frequency (%)
Particle Size (nm)
d=4,89±0,54 nm
ZnO-7
Frequency (%)
Particle Size (nm)

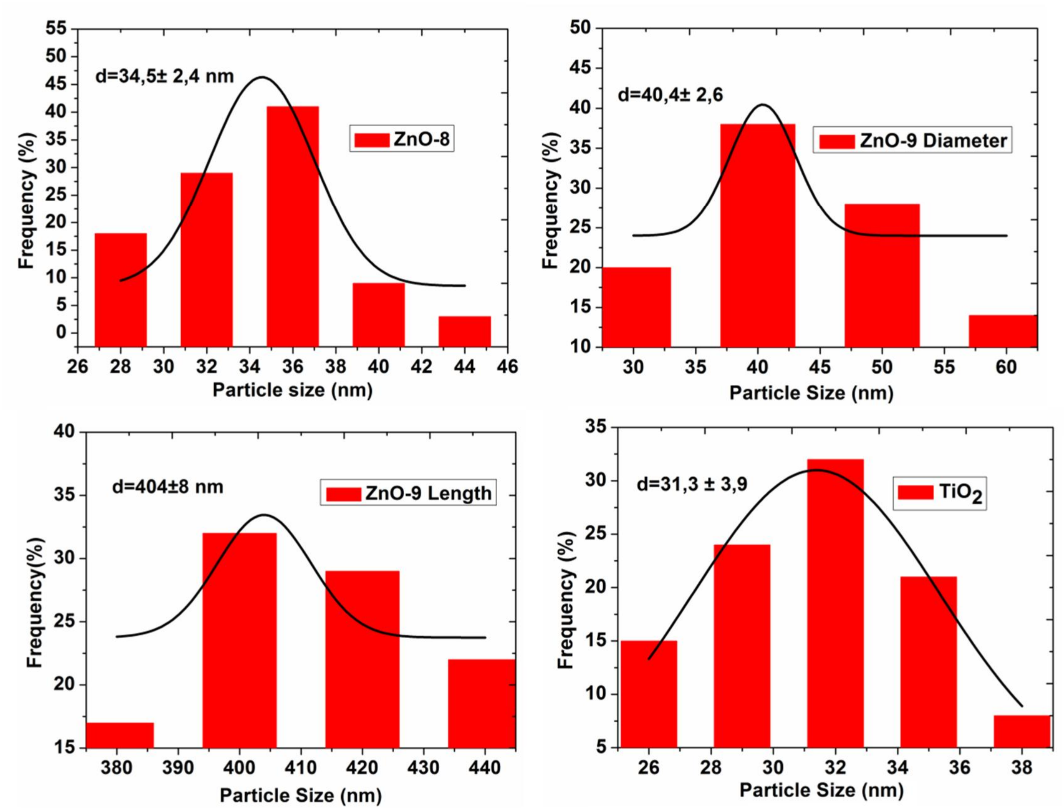

Figure S1. Size distribution charts of the ENs. Transmission electron microscopy (TEM) was used to investigate the size of the ENs. Hundred particles were counted for each sample. Size distribution charts presented show the particle size frequencies, an average diameter and a calculated statistical standard deviation acquired with non-linear fitting of the data for each sample.
