# Supplementary material for: Gene Expression Profiling of Immune-Competent Human Cells Exposed to Engineered Zinc Oxide or Titanium Dioxide Nanoparticles
Source: PLoS One. 2013 Jul 22;8(7):e68415. doi: 10.1371/journal.pone.0068415 (PMC3718780; doi:10.1371/journal.pone.0068415)
Supplement: Figure S2 — TEM analysis of ZnO nanoparticles. TEM micrographs of A) ZnO-1, B) ZnO-2, C) ZnO-3, D) ZnO-4, E) ZnO-5, F) ZnO-6, G) ZnO-7, H) ZnO-8, I) ZnO-9 and J) TiO2. (PDF) [file pone.0068415.s002.pdf]

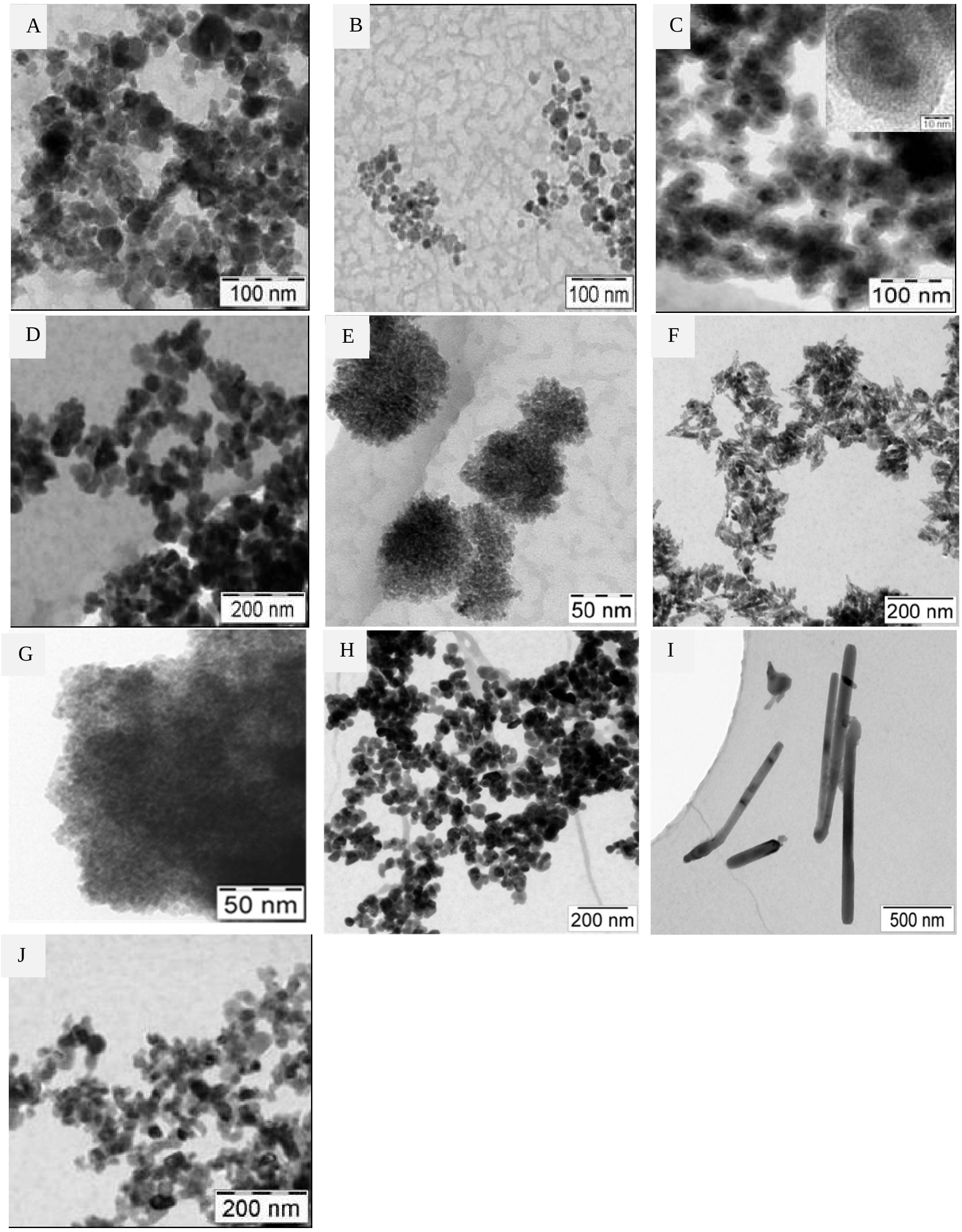


**Figure S2. TEM analysis of ZnO nanoparticles.** TEM micrographs of A) ZnO-1, B) ZnO-2, C) ZnO-3, D) ZnO-4, E) ZnO-5, F) ZnO-6, G) ZnO-7, H) ZnO-8, I) ZnO-9 and J) $TiO_2$.
