# Supplementary material for: Gene Expression Profiling of Immune-Competent Human Cells Exposed to Engineered Zinc Oxide or Titanium Dioxide Nanoparticles
Source: PLoS One. 2013 Jul 22;8(7):e68415. doi: 10.1371/journal.pone.0068415 (PMC3718780; doi:10.1371/journal.pone.0068415)
Supplement: Figure S3 — XRD pattern of ZnO nanoparticles. XRD spectra for ZnO-5, ZnO-6, ZnO-7, ZnO-8 and ZnO-9. They all reveal the typical peaks of hexagonal wurtzite type crystal structure. XRD results for ZnO-1, ZnO-2, ZnO-3, ZnO-4 and TiO2 have been presented in Buerki-Thurnherr et al. [7]. (PDF) [file pone.0068415.s003.pdf]

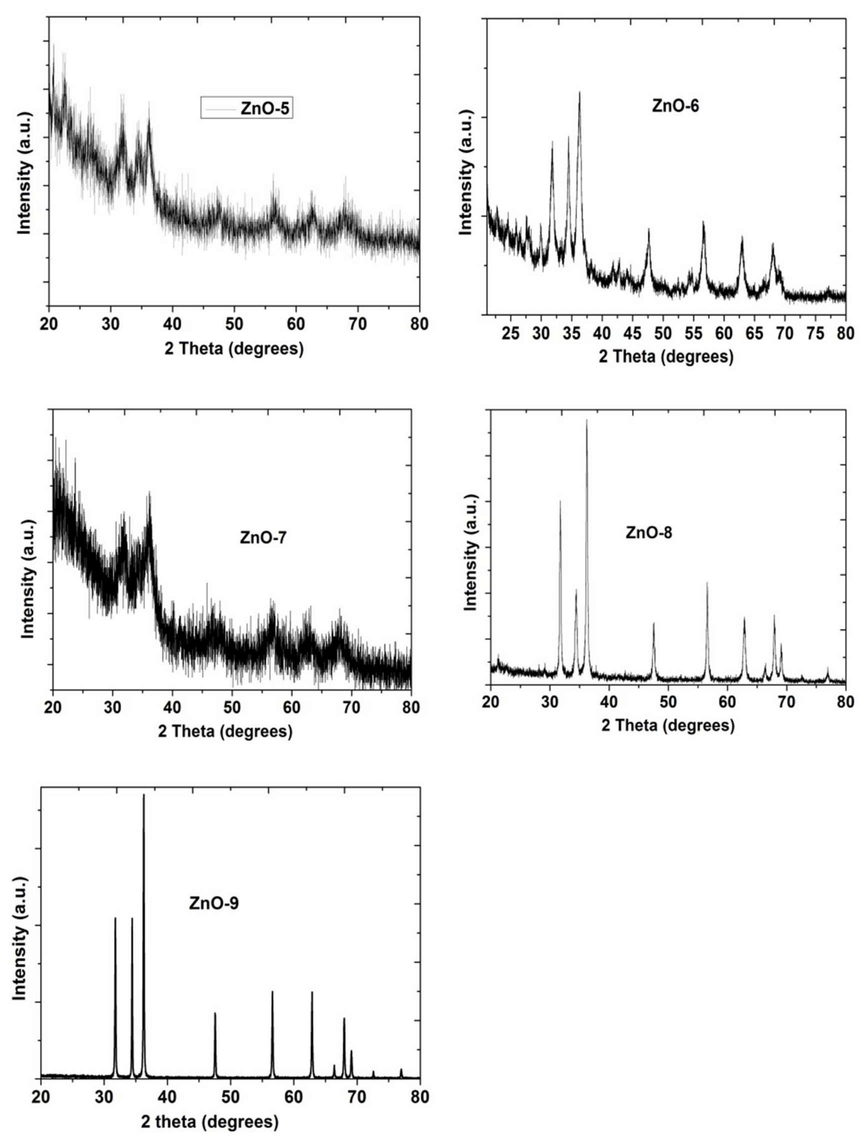

Figure S3. XRD pattern of ZnO nanoparticles. XRD spectra for ZnO-5, ZnO-6, ZnO-7, ZnO-8 and ZnO-9. They all reveal the typical peaks of hexagonal wurtzite type crystal structure. XRD results for ZnO-1, ZnO-2, ZnO-3, ZnO-4 and TiO_2 have been presented in Buerki-Thurnherr *et al.* [7].
