# Supplementary material for: Gene Expression Profiling of Immune-Competent Human Cells Exposed to Engineered Zinc Oxide or Titanium Dioxide Nanoparticles
Source: PLoS One. 2013 Jul 22;8(7):e68415. doi: 10.1371/journal.pone.0068415 (PMC3718780; doi:10.1371/journal.pone.0068415)
Supplement: Figure S4 — Schematic presentation of the experimental set-up for Illumina microarray hybridizations. HMDM samples are used as an example in the figure. Similar experiments were performed with MDDC and Jurkat cells. The red dotted arrows indicate the basic comparisons done for the microarray data. All experiments were repeated three times with independent cell cultures and analyzed with Illumina Sentrix HumanHT-12 Expression BeadChips. The samples marked with blue boxes were hybridized also to Affymetrix GeneChip Human Genome U219 array plates to validate the observations done with the Illumina arrays (Figure S9). Cell images from Biomedical PowerPoint Toolkit Suite (www.motifolio.com) were utilized in the figure preparation. (PDF) [file pone.0068415.s004.pdf]

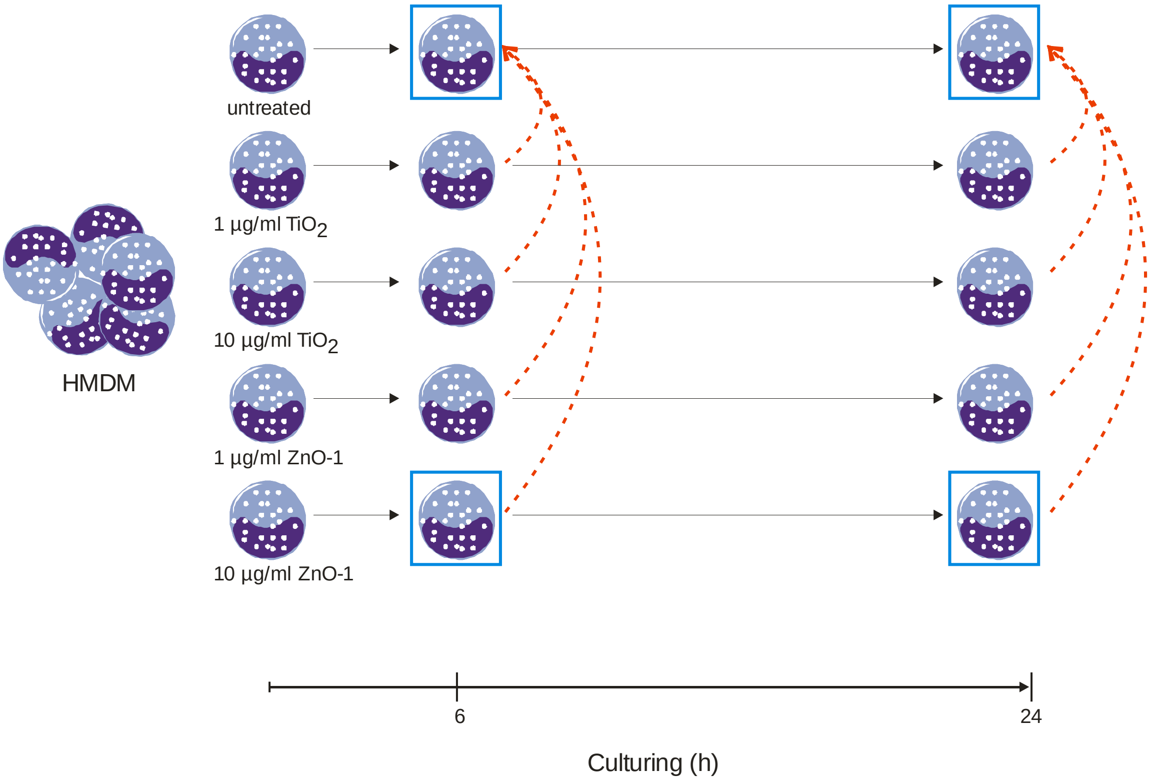

Figure S4. Schematic presentation of the experimental set-up for Illumina microarray hybridizations. HMDM samples are used as an example in the figure. Similar experiments were performed with MDDC and Jurkat cells. The red dotted arrows indicate the basic comparisons done for the microarray data. All experiments were repeated three times with independent cell cultures and analyzed with Illumina Sentrix HumanHT-12 Expression BeadChips. The samples marked with blue boxes were hybridized also to Affymetrix GeneChip Human Genome U219 array plates to validate the observations done with the Illumina arrays (**Figure S9**). Cell images from Biomedical PowerPoint Toolkit Suite (www.motifolio.com) were utilized in the figure preparation.
