# Supplementary material for: Gene Expression Profiling of Immune-Competent Human Cells Exposed to Engineered Zinc Oxide or Titanium Dioxide Nanoparticles
Source: PLoS One. 2013 Jul 22;8(7):e68415. doi: 10.1371/journal.pone.0068415 (PMC3718780; doi:10.1371/journal.pone.0068415)
Supplement: Figure S5 — MTT analysis of HMDM. Toxicity of ZnO-1 and TiO2 ENs were assessed with MTT assay. ZnO-1 caused dose-dependent loss of cell viability whereas TiO2 was inert. The figure shows an average and the standard deviation of three independent cultures. Similar trends in toxicity were obtained using MDDC [23] and the Jurkat cells [7]. The cell viability of HMDM, MDDC and Jurkat cells with 10 µg/ml of ZnO-1 at 24 h timepoint was around 55%, 70%, and 70%, respectively. (PDF) [file pone.0068415.s005.pdf]

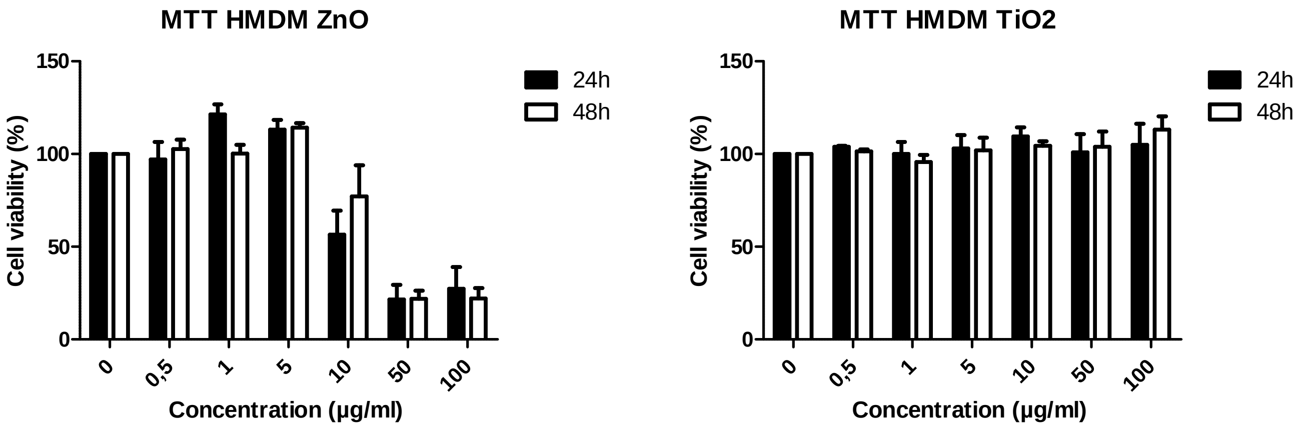

Figure S5. MTT analysis of HMDM. Toxicity of ZnO-1 and TiO_2 ENs were assessed with MTT assay. ZnO-1 caused dose-dependent loss of cell viability whereas TiO_2 was inert. The figure shows an average and the standard deviation of three independent cultures. Similar trends in toxicity were obtained using MDDC [23] and the Jurkat cells [7]. The cell viability of HMDM, MDDC and Jurkat cells with 10 µg/ml of ZnO-1 at 24 h timepoint was around 55%, 70%, and 70%, respectively.
