# Supplementary material for: Gene Expression Profiling of Immune-Competent Human Cells Exposed to Engineered Zinc Oxide or Titanium Dioxide Nanoparticles
Source: PLoS One. 2013 Jul 22;8(7):e68415. doi: 10.1371/journal.pone.0068415 (PMC3718780; doi:10.1371/journal.pone.0068415)
Supplement: Figure S6 — ZnO-1 and TiO2 induced gene expression in HMDM, MDDC and Jurkat cells. The number of genes having on average at least 1.5-fold difference in expression between the nanoparticle exposed and the untreated control cells in the whole-genome microarray analysis with Illumina Sentrix HumanHT-12 Expression BeadChips. The cell type, timepoint and the doses are indicated in the figure. The red bars represent the genes, which were upregulated after nanoparticle treatment and the green the downregulated ones. Three replicates of each treatment were used in the analysis. (PDF) [file pone.0068415.s006.pdf]

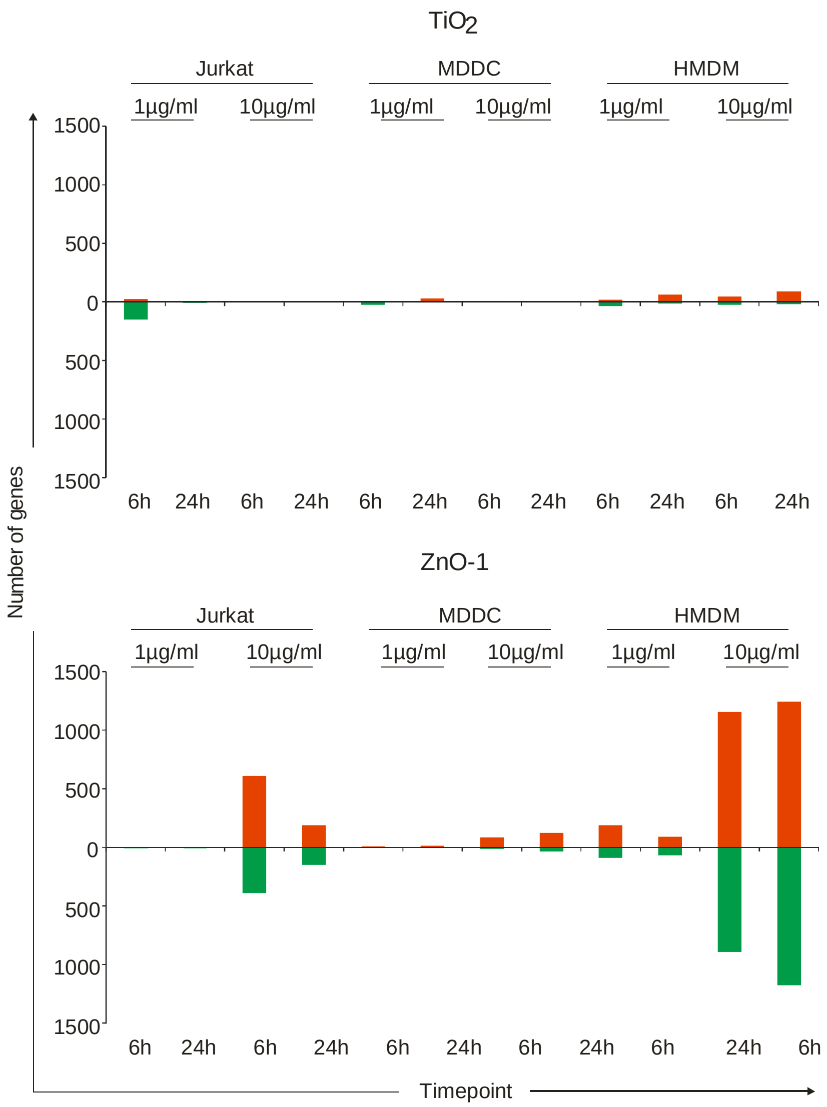

Figure S6. ZnO-1 and TiO_2 induced gene expression in HMDM, MDDC and Jurkat cells. The number of genes having on average at least 1.5-fold difference in expression between the nanoparticle exposed and the untreated control cells in the whole-genome microarray analysis with Illumina Sentrix HumanHT-12 Expression BeadChips. The cell type, timepoint and the doses are indicated in the figure. The red bars represent the genes, which were upregulated after nanoparticle treatment and the green the downregulated ones. Three replicates of each treatment were used in the analysis.
