# Supplementary material for: Gene Expression Profiling of Immune-Competent Human Cells Exposed to Engineered Zinc Oxide or Titanium Dioxide Nanoparticles
Source: PLoS One. 2013 Jul 22;8(7):e68415. doi: 10.1371/journal.pone.0068415 (PMC3718780; doi:10.1371/journal.pone.0068415)
Supplement: Figure S7 — Expression of the metallothioneins validated with RT-PCR in response to ZnO-1 and TiO2 nanoparticles. Figure shows the normalized log2-transformed average expression levels in untreated control MDDC, HMDM and Jurkat cells and the expression after exposure of the cells with 10 µg/ml of ZnO-1 or 10 µg/ml of TiO2 at 6 and 24 h of culture. The data is from three replicate experiments. (PDF) [file pone.0068415.s007.pdf]

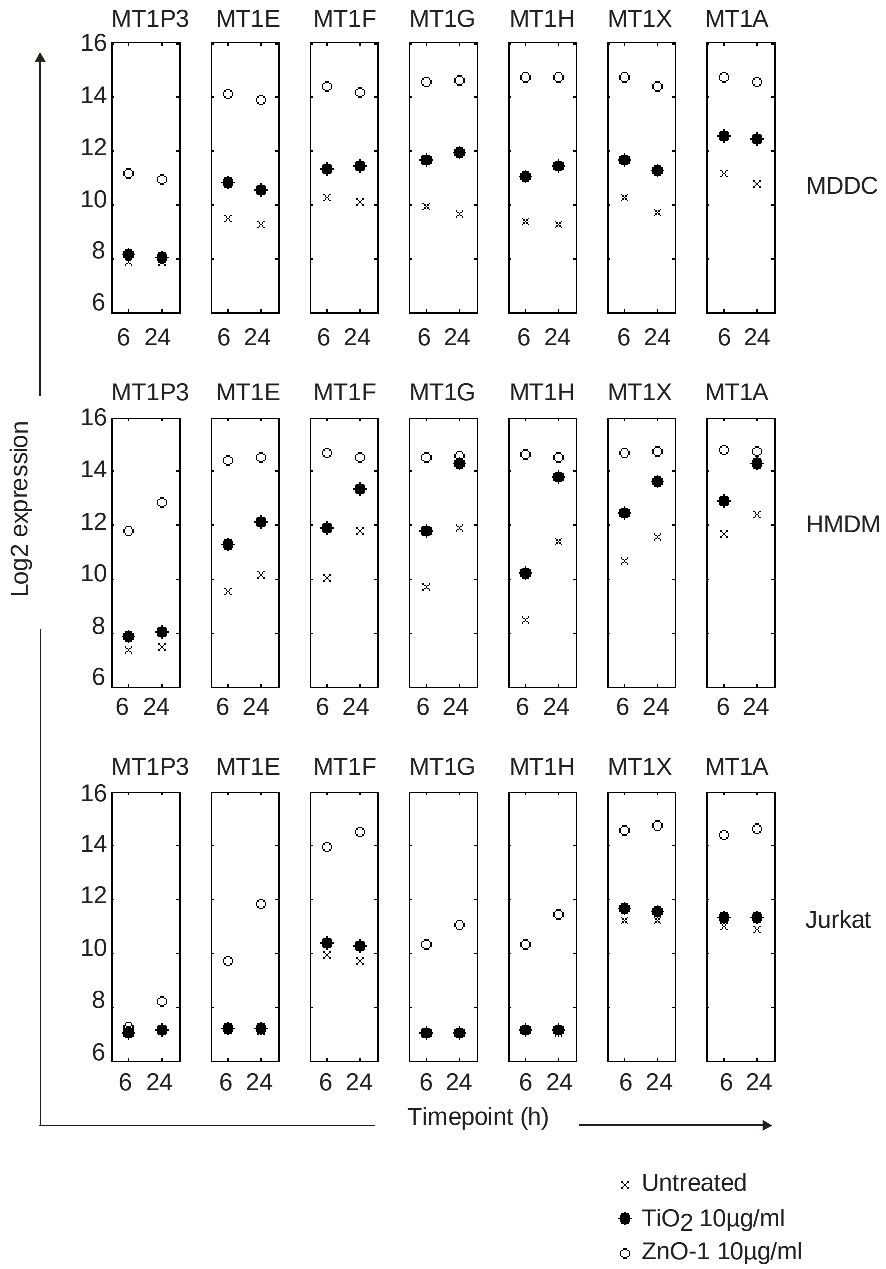

Figure S7. Expression of the metallothioneins validated with RT-PCR in response to ZnO-1 and TiO_2 nanoparticles. Figure shows the normalized log2-transformed average expression levels in untreated control MDDC, HMDM and Jurkat cells and the expression after exposure of the cells with 10 µg/ml of ZnO-1 or 10 µg/ml of TiO_2 at 6 and 24 h of culture. The data is from three replicate experiments.
