# Supplementary material for: Gene Expression Profiling of Immune-Competent Human Cells Exposed to Engineered Zinc Oxide or Titanium Dioxide Nanoparticles
Source: PLoS One. 2013 Jul 22;8(7):e68415. doi: 10.1371/journal.pone.0068415 (PMC3718780; doi:10.1371/journal.pone.0068415)
Supplement: Figure S8 — Hierarchical clustering of the expression of the ZnO responsive genes reported by Moos et al . [10] among A) HMDM samples and B) Jurkat samples in Illumina Sentrix HumanHT-12 Expression BeadChip data after exposing the cells with 1 µg/ml or 10 µg/ml of ZnO-1 or TiO2 for 6 or 24 h in three replicate experiments (see Figure S4 and Table S3). The subclusters discussed in the main text are highlighted. The gene expression data for the differentially expressed genes is shown in Table S4. (PDF) [file pone.0068415.s008.pdf]

A

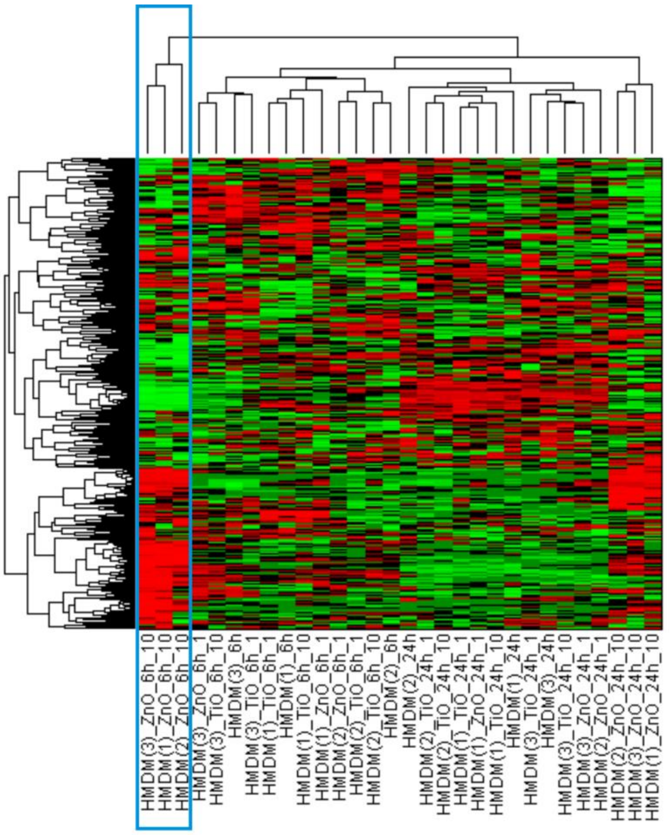
HMDM(3)_ZnO_6h_10
HMDM(1)_ZnO_6h_10
HMDM(2)_ZnO_6h_10
HMDM(3)_ZnO_6h_1
HMDM(3)_TiO_6h_10
HMDM(3)_6h
HMDM(3)_TiO_6h_1
HMDM(1)_TiO_6h_1
HMDM(1)_6h
HMDM(1)_TiO_6h_10
HMDM(1)_ZnO_6h_1
HMDM(2)_ZnO_6h_1
HMDM(2)_TiO_6h_1
HMDM(2)_TiO_6h_10
HMDM(2)_6h
HMDM(2)_24h
HMDM(2)_TiO_24h_1
HMDM(2)_TiO_24h_10
HMDM(1)_TiO_24h_1
HMDM(1)_ZnO_24h_1
HMDM(1)_TiO_24h_10
HMDM(1)_24h
HMDM(3)_TiO_24h_1
HMDM(3)_24h
HMDM(3)_TiO_24h_10
HMDM(3)_ZnO_24h_1
HMDM(2)_ZnO_24h_1
HMDM(2)_ZnO_24h_10
HMDM(3)_ZnO_24h_10
HMDM(1)_ZnO_24h_10

B

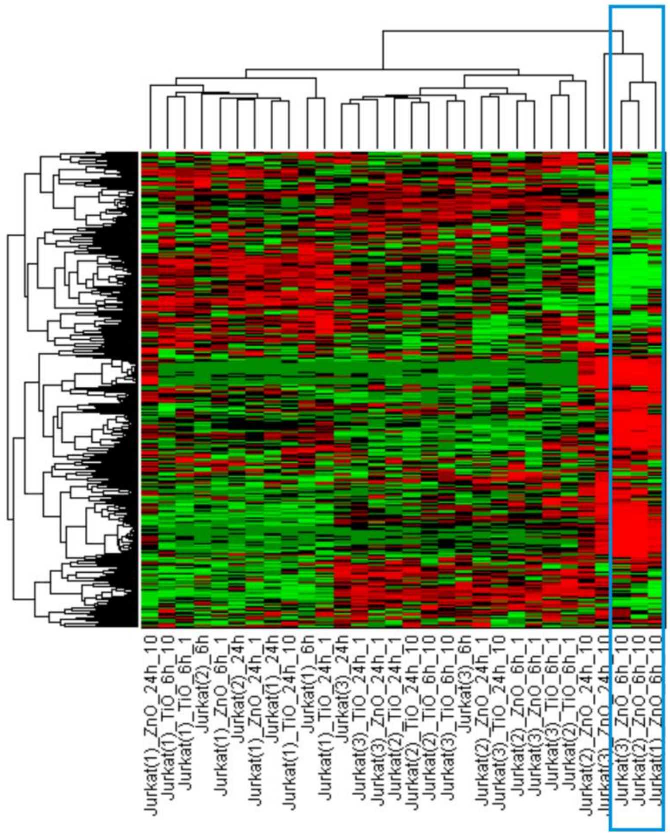
Jurkat(1)_ZnO_24h_10
Jurkat(1)_TiO_6h_10
Jurkat(1)_TiO_6h_1
Jurkat(2)_6h
Jurkat(1)_ZnO_6h_1
Jurkat(2)_24h
Jurkat(1)_ZnO_24h_1
Jurkat(1)_24h
Jurkat(1)_TiO_24h_10
Jurkat(1)_6h
Jurkat(1)_TiO_24h_1
Jurkat(3)_24h
Jurkat(3)_TiO_24h_1
Jurkat(3)_ZnO_24h_1
Jurkat(2)_TiO_24h_1
Jurkat(2)_TiO_24h_10
Jurkat(2)_TiO_6h_10
Jurkat(3)_TiO_6h_10
Jurkat(3)_6h
Jurkat(2)_ZnO_24h_1
Jurkat(3)_TiO_24h_10
Jurkat(2)_ZnO_6h_1
Jurkat(3)_ZnO_6h_1
Jurkat(3)_TiO_6h_1
Jurkat(2)_TiO_6h_1
Jurkat(2)_ZnO_24h_10
Jurkat(3)_ZnO_24h_10
Jurkat(3)_ZnO_6h_10
Jurkat(2)_ZnO_6h_10
Jurkat(1)_ZnO_6h_10

Figure S8. Hierarchical clustering of the expression of the ZnO responsive genes reported by Moos *et al.* [10] among A) HMDM samples and B) Jurkat samples in Illumina Sentrix HumanHT-12 Expression BeadChip data after exposing the cells with 1 µg/ml or 10 µg/ml of ZnO-1 or TiO_2 for 6 or 24 h in three replicate experiments (see **Figure S4** and **Table S3**). The subclusters discussed in the main text are highlighted. The gene expression data for the differentially expressed genes is shown in **Table S4**.
