# Supplementary material for: Gene Expression Profiling of Immune-Competent Human Cells Exposed to Engineered Zinc Oxide or Titanium Dioxide Nanoparticles
Source: PLoS One. 2013 Jul 22;8(7):e68415. doi: 10.1371/journal.pone.0068415 (PMC3718780; doi:10.1371/journal.pone.0068415)
Supplement: Figure S9 — Schematic presentation of the experimental set-up for Affymetrix GeneChip Human Genome U219 array hybridizations. MDDC samples from four donors treated for 24 h with 10 µg/ml of ZnO-1 or cultured without exposure were hybridized to Affymetrix arrays. At the same time ZnO-1 (10 µg/ml) or untreated control cells cultured for 6 or 24 h from three replicate experiments of MDDC, HMDM and Jurkat cells previously analyzed with Illumina arrays were processed for array validation (Figure S4, Table S3). In addition, samples from Jurkat cells treated with ZnO-2, ZnO-3, ZnO-5 or ZnO-9 (10 µg/ml, 24 h), or ZnO-4 (10, 25, 50 or 100 µg/ml, 24h) were analyzed. The red dotted arrows indicate the basic comparisons done for the microarray data. Cell images from Biomedical PowerPoint Toolkit Suite (www.motifolio.com) were utilized in the figure preparation. (PDF) [file pone.0068415.s009.pdf]

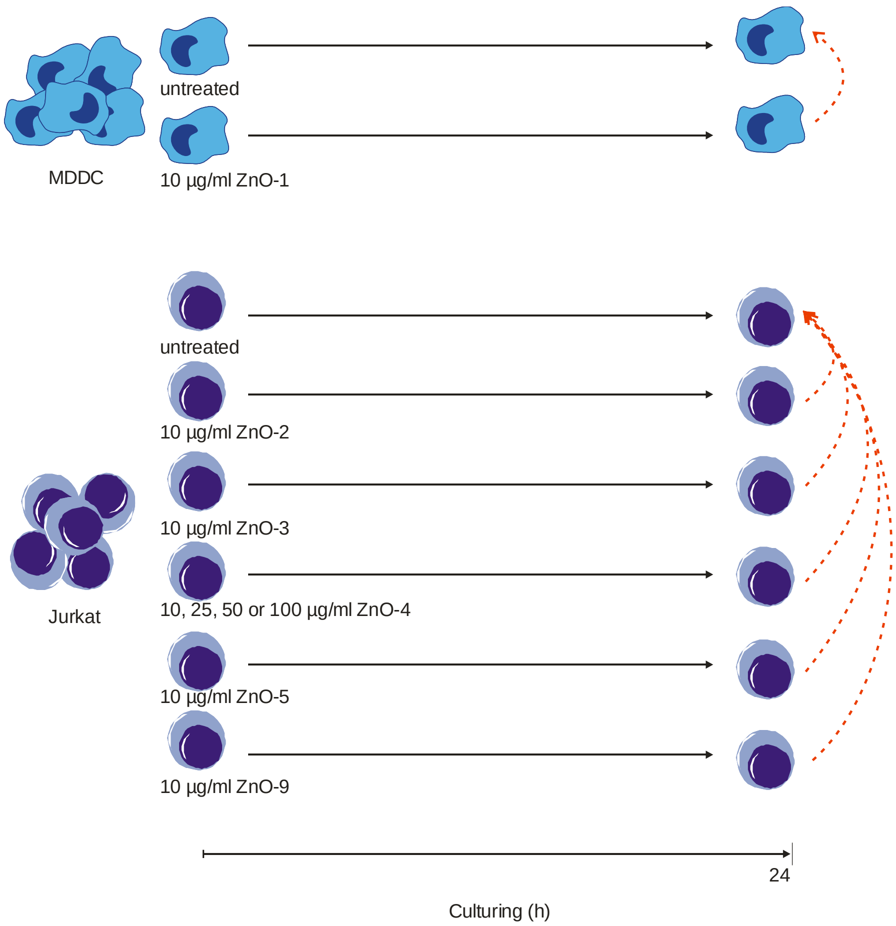

Figure S9. Schematic presentation of the experimental set-up for Affymetrix GeneChip Human Genome U219 array hybridizations. MDDC samples from four donors treated for 24 h with 10 µg/ml of ZnO-1 or cultured without exposure were hybridized to Affymetrix arrays. At the same time ZnO-1 (10 µg/ml) or untreated control cells cultured for 6 or 24 h from three replicate experiments of MDDC, HMDM and Jurkat cells previously analyzed with Illumina arrays were processed for array validation (**Figure S4**, **Table S3**). In addition, samples from Jurkat cells treated with ZnO-2, ZnO-3, ZnO-5 or ZnO-9 (10 µg/ml, 24 h), or ZnO-4 (10, 25, 50 or 100 µg/ml, 24h) were analyzed. The red dotted arrows indicate the basic comparisons done for the microarray data. Cell images from Biomedical PowerPoint Toolkit Suite (www.motifolio.com) were utilized in the figure preparation.
