# Supplementary material for: Gene Expression Profiling of Immune-Competent Human Cells Exposed to Engineered Zinc Oxide or Titanium Dioxide Nanoparticles
Source: PLoS One. 2013 Jul 22;8(7):e68415. doi: 10.1371/journal.pone.0068415 (PMC3718780; doi:10.1371/journal.pone.0068415)
Supplement: Figure S10 — ZnO-1 response in MDDCs. A) Hierarchical clustering of the ZnO-1 (10 µg/ml) treated, or the untreated control MDDC samples using correlation distance and complete linkage in clustering. Seven different donors were used in the analysis. The gene expressions were measured with Affymetrix GeneChip Human Genome U219 array plates. The samples from the donors 1–3 are the same samples hybridized to Illumina Sentrix HumanHT-12 v3 Expression BeadChips, thus being platform independent technical replicates. The name of the sample is formed by combining the information about cell type, number of replicate in brackets, treatment and timepoint. B) The overlap of the detected statistically (FDR≤0.05 and absolute FC≥1.5) differentially expressed genes between the ZnO-1 treated cells and the untreated control cells at the indicated timepoints measured either with Affymetrix GeneChip Human Genome U219 array plates or Illumina Sentrix HumanHT-12 v3 Expression BeadChips. The figure was drawn with VENNY [59]. C) Hierarchical clustering results of all the samples analyzed with Illumina microarrays; HMDM, MDDC or Jurkat cells exposed with 1µg/ml or 10µg/ml of ZnO-1 or TiO2 for 6 h or 24 h, (see Figure S4 and Table S3) by using correlation distance with complete linkage. (PDF) [file pone.0068415.s010.pdf]

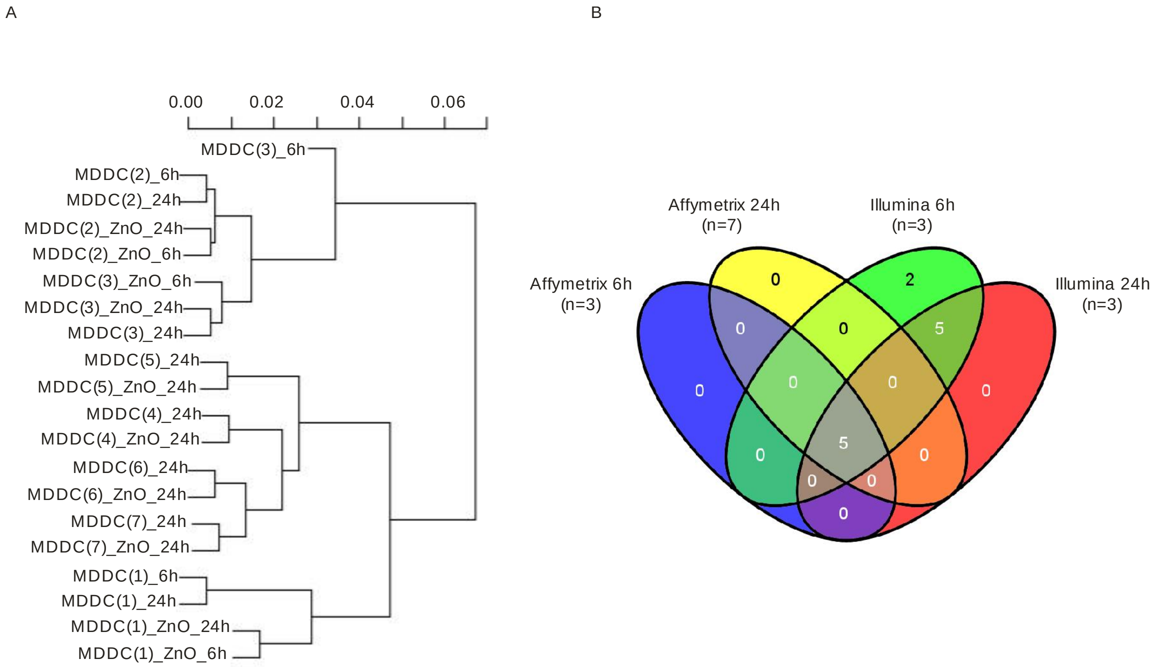

Figure S10. ZnO-1 response in MDDCs. A) Hierarchical clustering of the ZnO-1 (10 µg/ml) treated, or the untreated control MDDC samples using correlation distance and complete linkage in clustering. Seven different donors were used in the analysis. The gene expressions were measured with Affymetrix GeneChip Human Genome U219 array plates. The samples from the donors 1-3 are the same samples hybridized to Illumina Sentrix HumanHT-12 v3 Expression BeadChips, thus being platform independent technical replicates. The name of the sample is formed by combining the information about cell type, number of replicate in brackets, treatment and timepoint. B) The overlap of the detected statistically (FDR≤0.05 and absolute FC≥1.5) differentially expressed genes between the ZnO-1 treated cells and the untreated control cells at the indicated timepoints measured either with Affymetrix GeneChip Human Genome U219 array plates or Illumina Sentrix HumanHT-12 v3 Expression BeadChips. The figure was drawn with VENNY [59]. C) Hierarchical clustering results of all the samples analyzed with Illumina microarrays; HMDM, MDDC or

Jurkat cells exposed with 1µg/ml or 10µg/ml of ZnO-1 or TiO_2 for 6 h or 24 h, (see **Figure S4** and **Table S3**) by using correlation distance with complete linkage.

c

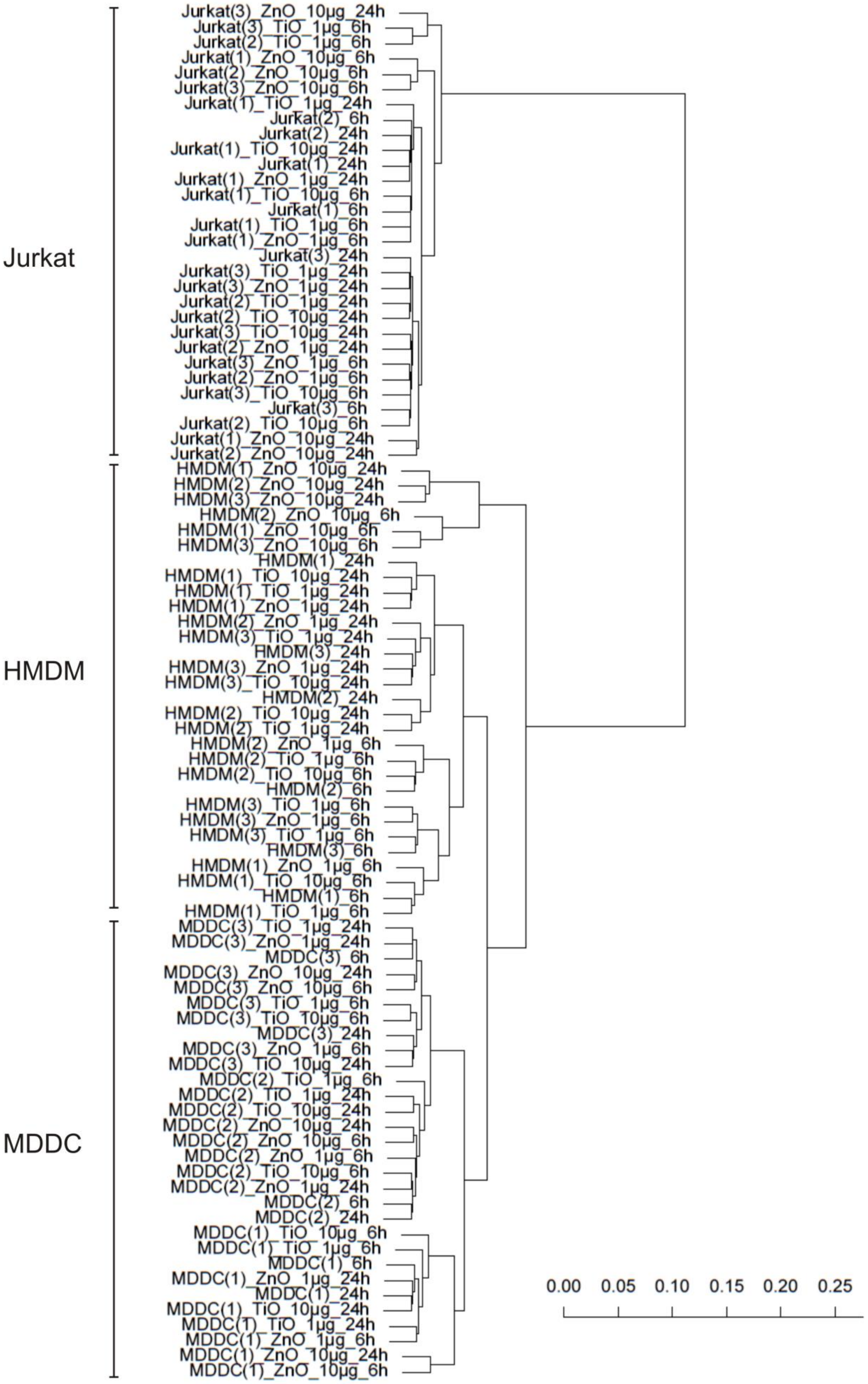
Jurkat
HMDM
MDDC
Jurkat(3)_ZnO_10µg_24h
Jurkat(3)_TiO_1µg_6h
Jurkat(2)_TiO_1µg_6h
Jurkat(1)_ZnO_10µg_6h
Jurkat(2)_ZnO_10µg_6h
Jurkat(3)_ZnO_10µg_6h
Jurkat(1)_TiO_1µg_24h
Jurkat(2)_6h
Jurkat(2)_24h
Jurkat(1)_TiO_10µg_24h
Jurkat(1)_24h
Jurkat(1)_ZnO_1µg_24h
Jurkat(1)_TiO_10µg_6h
Jurkat(1)_6h
Jurkat(1)_TiO_1µg_6h
Jurkat(1)_ZnO_1µg_6h
Jurkat(3)_24h
Jurkat(3)_TiO_1µg_24h
Jurkat(3)_ZnO_1µg_24h
Jurkat(2)_TiO_1µg_24h
Jurkat(2)_TiO_10µg_24h
Jurkat(3)_TiO_10µg_24h
Jurkat(2)_ZnO_1µg_24h
Jurkat(3)_ZnO_1µg_6h
Jurkat(2)_ZnO_1µg_6h
Jurkat(3)_TiO_10µg_6h
Jurkat(3)_6h
Jurkat(2)_TiO_10µg_6h
Jurkat(1)_ZnO_10µg_24h
Jurkat(2)_ZnO_10µg_24h
HMDM(1)_ZnO_10µg_24h
HMDM(2)_ZnO_10µg_24h
HMDM(3)_ZnO_10µg_24h
HMDM(2)_ZnO_10µg_6h
HMDM(1)_ZnO_10µg_6h
HMDM(3)_ZnO_10µg_6h
HMDM(1)_24h
HMDM(1)_TiO_10µg_24h
HMDM(1)_TiO_1µg_24h
HMDM(1)_ZnO_1µg_24h
HMDM(2)_ZnO_1µg_24h
HMDM(3)_TiO_1µg_24h
HMDM(3)_24h
HMDM(3)_ZnO_1µg_24h
HMDM(3)_TiO_10µg_24h
HMDM(2)_24h
HMDM(2)_TiO_10µg_24h
HMDM(2)_TiO_1µg_24h
HMDM(2)_ZnO_1µg_6h
HMDM(2)_TiO_1µg_6h
HMDM(2)_TiO_10µg_6h
HMDM(2)_6h
HMDM(3)_TiO_1µg_6h
HMDM(3)_ZnO_1µg_6h
HMDM(3)_TiO_1µg_6h
HMDM(3)_6h
HMDM(1)_ZnO_1µg_6h
HMDM(1)_TiO_10µg_6h
HMDM(1)_6h
HMDM(1)_TiO_1µg_6h
MDDC(3)_TiO_1µg_24h
MDDC(3)_ZnO_1µg_24h
MDDC(3)_6h
MDDC(3)_ZnO_10µg_24h
MDDC(3)_ZnO_10µg_6h
MDDC(3)_TiO_1µg_6h
MDDC(3)_TiO_10µg_6h
MDDC(3)_24h
MDDC(3)_ZnO_1µg_6h
MDDC(3)_TiO_10µg_24h
MDDC(2)_TiO_1µg_6h
MDDC(2)_TiO_1µg_24h
MDDC(2)_TiO_10µg_24h
MDDC(2)_ZnO_10µg_24h
MDDC(2)_ZnO_10µg_6h
MDDC(2)_ZnO_1µg_6h
MDDC(2)_TiO_10µg_6h
MDDC(2)_ZnO_1µg_24h
MDDC(2)_6h
MDDC(2)_24h
MDDC(1)_TiO_10µg_6h
MDDC(1)_TiO_1µg_6h
MDDC(1)_6h
MDDC(1)_ZnO_1µg_24h
MDDC(1)_24h
MDDC(1)_TiO_10µg_24h
MDDC(1)_TiO_1µg_24h
MDDC(1)_ZnO_1µg_6h
MDDC(1)_ZnO_10µg_24h
MDDC(1)_ZnO_10µg_6h
0.00 0.05 0.10 0.15 0.20 0.25
