# Supplementary material for: Gene Expression Profiling of Immune-Competent Human Cells Exposed to Engineered Zinc Oxide or Titanium Dioxide Nanoparticles
Source: PLoS One. 2013 Jul 22;8(7):e68415. doi: 10.1371/journal.pone.0068415 (PMC3718780; doi:10.1371/journal.pone.0068415)
Supplement: Figure S11 — Donor or replicate culture -specific responses to ZnO-1. Venn diagrams of the differentially expressed genes in MDDC, HMDM and Jurkat cells (FC>1.5 or <−1.5) in each donor or replicate experiment at 6 h and 24 h timepoints after exposure to 10 µg/ml of ZnO-1 compared to the corresponding untreated control cells analyzed with Illumina Sentrix HumanHT-12 Expression BeadChips. (PDF) [file pone.0068415.s011.pdf]

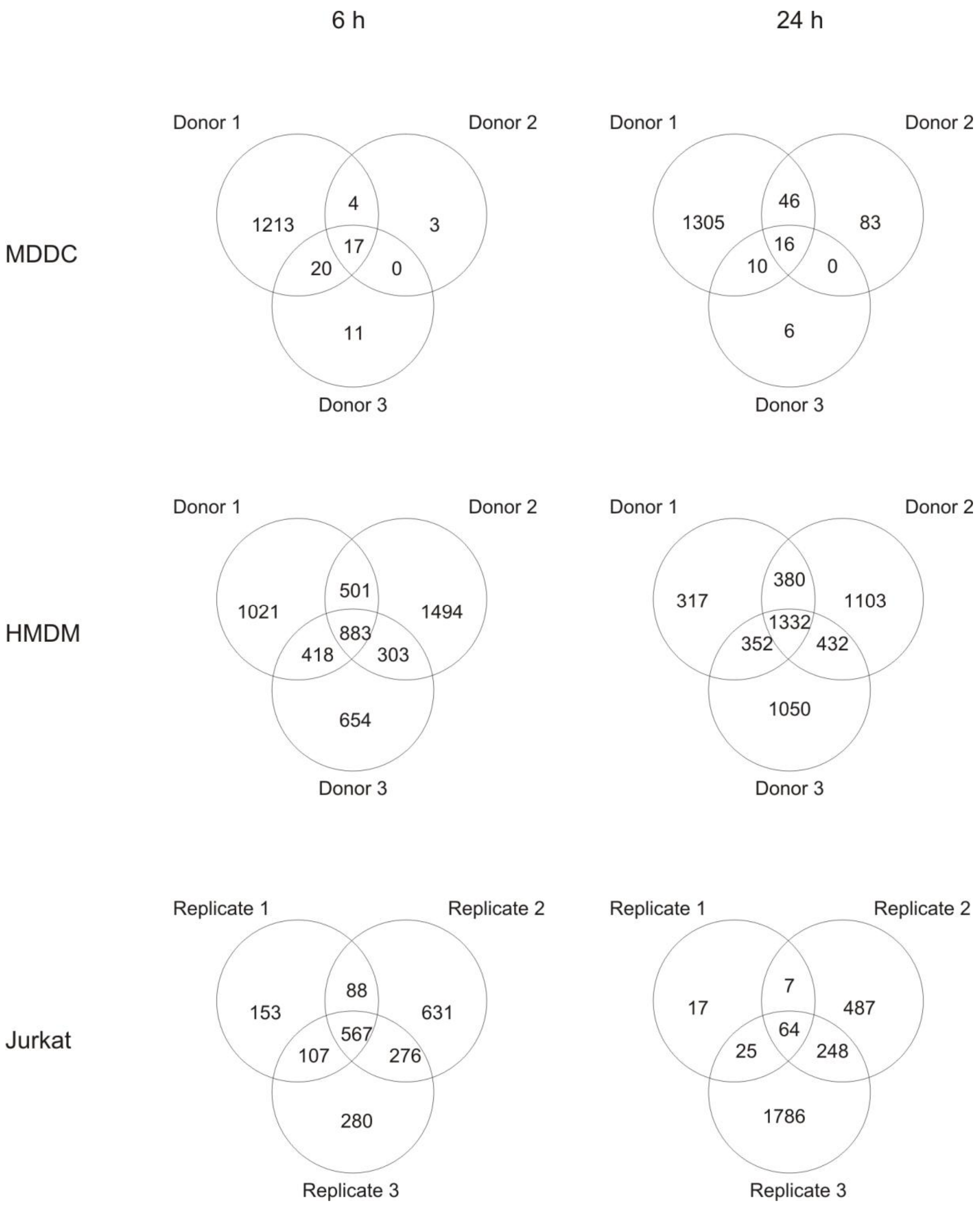

Figure S11. Donor or replicate culture -specific responses to ZnO-1. Venn diagrams of the differentially expressed genes in MDDC, HMDM and Jurkat cells (FC>1.5 or <-1.5) in each donor or replicate experiment at 6 h and 24 h timepoints after exposure to 10 µg/ml of ZnO-1 compared to the corresponding untreated control cells analyzed with Illumina Sentrix HumanHT-12 Expression BeadChips.
