# Supplementary material for: Gene Expression Profiling of Immune-Competent Human Cells Exposed to Engineered Zinc Oxide or Titanium Dioxide Nanoparticles
Source: PLoS One. 2013 Jul 22;8(7):e68415. doi: 10.1371/journal.pone.0068415 (PMC3718780; doi:10.1371/journal.pone.0068415)
Supplement: Figure S12 — Hierarchical clustering of the samples analyzed with Affymetrix GeneChip Human Genome U219 array plates. The data is from three replicate experiments of Jurkat cells exposed with 10 µg/ml of ZnO-2, ZnO-3, ZnO-5 or ZnO-9 for 24 h; or 10 µg/ml, 25 µg/ml, 50 µg/ml or 100 µg/ml of ZnO-4 for 24 h; HMDM, MDDC or Jurkat cells exposed with 10 µg/ml of ZnO-1 for 6 h and 24 h, and 4 replicates experiments of MDDC exposed with 10 µg/ml of ZnO-1 for 24 h (see Figure S4, Figure S9 and Table S3). The clustering was done by using correlation distance with complete linkage. (PDF) [file pone.0068415.s012.pdf]

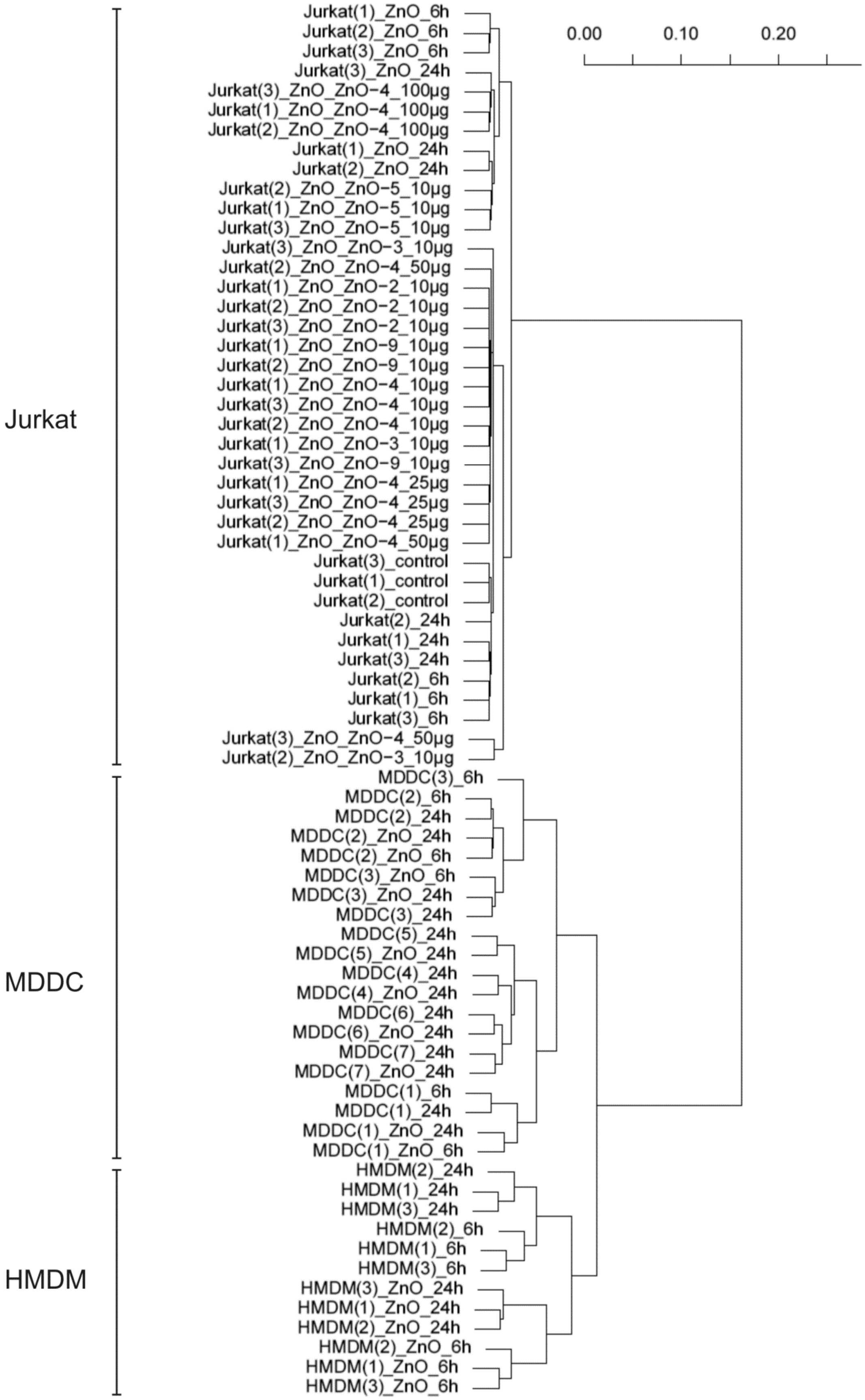
0.00
0.10
0.20
Jurkat
MDDC
HMDM
Jurkat(1)_ZnO_6h
Jurkat(2)_ZnO_6h
Jurkat(3)_ZnO_6h
Jurkat(3)_ZnO_24h
Jurkat(3)_ZnO_ZnO−4_100µg
Jurkat(1)_ZnO_ZnO−4_100µg
Jurkat(2)_ZnO_ZnO−4_100µg
Jurkat(1)_ZnO_24h
Jurkat(2)_ZnO_24h
Jurkat(2)_ZnO_ZnO−5_10µg
Jurkat(1)_ZnO_ZnO−5_10µg
Jurkat(3)_ZnO_ZnO−5_10µg
Jurkat(3)_ZnO_ZnO−3_10µg
Jurkat(2)_ZnO_ZnO−4_50µg
Jurkat(1)_ZnO_ZnO−2_10µg
Jurkat(2)_ZnO_ZnO−2_10µg
Jurkat(3)_ZnO_ZnO−2_10µg
Jurkat(1)_ZnO_ZnO−9_10µg
Jurkat(2)_ZnO_ZnO−9_10µg
Jurkat(1)_ZnO_ZnO−4_10µg
Jurkat(3)_ZnO_ZnO−4_10µg
Jurkat(2)_ZnO_ZnO−4_10µg
Jurkat(1)_ZnO_ZnO−3_10µg
Jurkat(3)_ZnO_ZnO−9_10µg
Jurkat(1)_ZnO_ZnO−4_25µg
Jurkat(3)_ZnO_ZnO−4_25µg
Jurkat(2)_ZnO_ZnO−4_25µg
Jurkat(1)_ZnO_ZnO−4_50µg
Jurkat(3)_control
Jurkat(1)_control
Jurkat(2)_control
Jurkat(2)_24h
Jurkat(1)_24h
Jurkat(3)_24h
Jurkat(2)_6h
Jurkat(1)_6h
Jurkat(3)_6h
Jurkat(3)_ZnO_ZnO−4_50µg
Jurkat(2)_ZnO_ZnO−3_10µg
MDDC(3)_6h
MDDC(2)_6h
MDDC(2)_24h
MDDC(2)_ZnO_24h
MDDC(2)_ZnO_6h
MDDC(3)_ZnO_6h
MDDC(3)_ZnO_24h
MDDC(3)_24h
MDDC(5)_24h
MDDC(5)_ZnO_24h
MDDC(4)_24h
MDDC(4)_ZnO_24h
MDDC(6)_24h
MDDC(6)_ZnO_24h
MDDC(7)_24h
MDDC(7)_ZnO_24h
MDDC(1)_6h
MDDC(1)_24h
MDDC(1)_ZnO_24h
MDDC(1)_ZnO_6h
HMDM(2)_24h
HMDM(1)_24h
HMDM(3)_24h
HMDM(2)_6h
HMDM(1)_6h
HMDM(3)_6h
HMDM(3)_ZnO_24h
HMDM(1)_ZnO_24h
HMDM(2)_ZnO_24h
HMDM(2)_ZnO_6h
HMDM(1)_ZnO_6h
HMDM(3)_ZnO_6h

Figure S12. Hierarchical clustering of the samples analyzed with Affymetrix GeneChip Human Genome U219 array plates. The data is from three replicate experiments of Jurkat cells exposed with 10 µg/ml of ZnO-2, ZnO-3, ZnO-5 or ZnO-9 for 24 h; or 10 µg/ml, 25 µg/ml, 50 µg/ml or 100 µg/ml of ZnO-4 for 24 h; HMDM, MDDC or Jurkat cells exposed with 10 µg/ml of ZnO-1 for 6 h and 24 h, and 4 replicates experiments of MDDC exposed with 10 µg/ml of ZnO-1 for 24 h (see **Figure S4**, **Figure S9** and **Table S3**). The clustering was done by using correlation distance with complete linkage.
