# Supplementary material for: Gene Expression Profiling of Immune-Competent Human Cells Exposed to Engineered Zinc Oxide or Titanium Dioxide Nanoparticles
Source: PLoS One. 2013 Jul 22;8(7):e68415. doi: 10.1371/journal.pone.0068415 (PMC3718780; doi:10.1371/journal.pone.0068415)
Supplement: Figure S13 — Hierarchical clustering of the expression of the zinc responsive genes reported by Cousins et al . [28] among A) HMDM samples and B) Jurkat samples in Illumina Sentrix HumanHT-12 Expression BeadChip data after exposing the cells with 1 µg/ml or 10 µg/ml of ZnO-1 or TiO2 for 6 or 24 h in three replicate experiments (see Figure S4 and Table S3). The subclusters separating different treatments are highlighted. The gene expression data for the differentially expressed genes is shown in Table S9. (PDF) [file pone.0068415.s013.pdf]

A

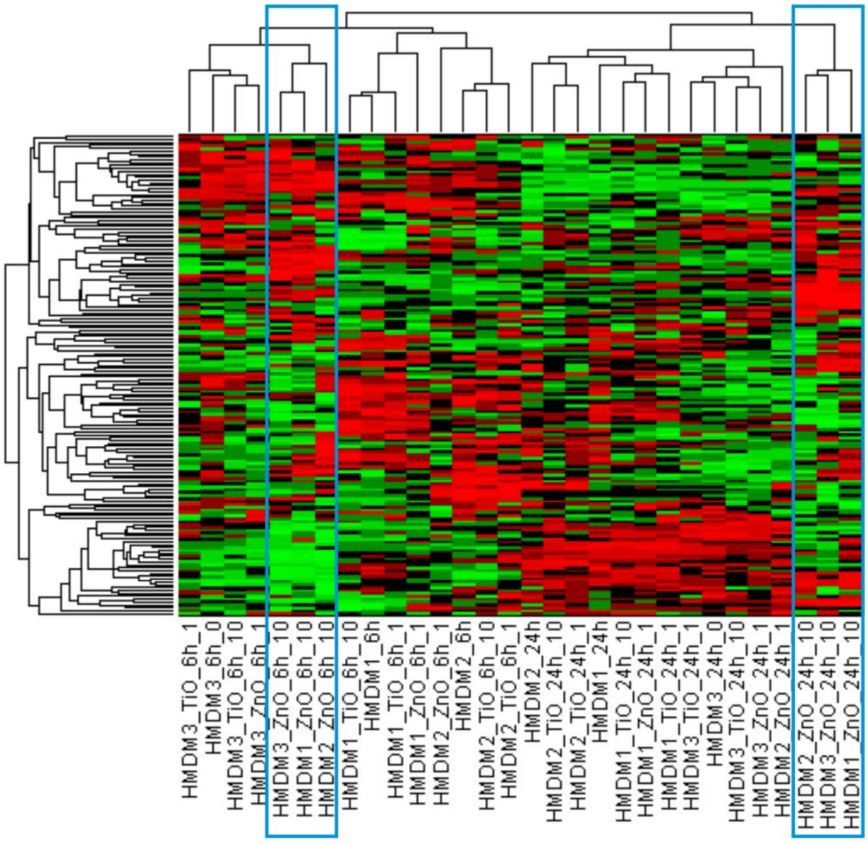
HMDM3_TiO_6h_1
HMDM3_6h_0
HMDM3_TiO_6h_10
HMDM3_ZnO_6h_1
HMDM3_ZnO_6h_10
HMDM1_ZnO_6h_10
HMDM2_ZnO_6h_10
HMDM1_TiO_6h_10
HMDM1_6h
HMDM1_TiO_6h_1
HMDM1_ZnO_6h_1
HMDM2_ZnO_6h_1
HMDM2_6h
HMDM2_TiO_6h_10
HMDM2_TiO_6h_1
HMDM2_24h
HMDM2_TiO_24h_10
HMDM2_TiO_24h_1
HMDM1_24h
HMDM1_TiO_24h_10
HMDM1_ZnO_24h_1
HMDM1_TiO_24h_1
HMDM3_TiO_24h_1
HMDM3_24h_0
HMDM3_TiO_24h_10
HMDM3_ZnO_24h_1
HMDM2_ZnO_24h_1
HMDM2_ZnO_24h_10
HMDM3_ZnO_24h_10
HMDM1_ZnO_24h_10

B

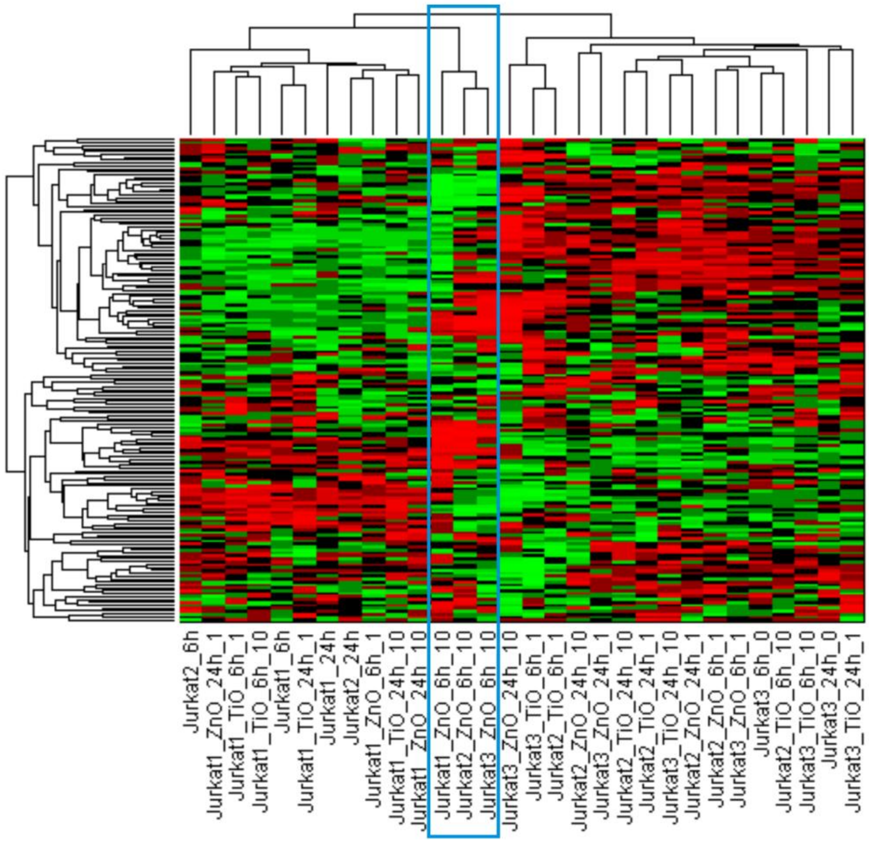
Jurkat2_6h
Jurkat1_ZnO_24h_1
Jurkat1_TiO_6h_1
Jurkat1_TiO_6h_10
Jurkat1_6h
Jurkat1_TiO_24h_1
Jurkat1_24h
Jurkat2_24h
Jurkat1_ZnO_6h_1
Jurkat1_TiO_24h_10
Jurkat1_ZnO_24h_10
Jurkat1_ZnO_6h_10
Jurkat2_ZnO_6h_10
Jurkat3_ZnO_6h_10
Jurkat3_ZnO_24h_10
Jurkat3_TiO_6h_1
Jurkat3_TiO_6h_10
Jurkat2_ZnO_24h_10
Jurkat3_ZnO_24h_1
Jurkat2_TiO_24h_1
Jurkat2_TiO_24h_10
Jurkat3_TiO_24h_10
Jurkat2_ZnO_24h_1
Jurkat2_ZnO_6h_1
Jurkat3_ZnO_6h_1
Jurkat3_6h_0
Jurkat2_TiO_6h_10
Jurkat3_TiO_6h_10
Jurkat3_24h_0
Jurkat3_TiO_24h_1

Figure S13. Hierarchical clustering of the expression of the zinc responsive genes reported by Cousins *et al*. [28] among A) HMDM samples and B) Jurkat samples in Illumina Sentrix HumanHT-12 Expression BeadChip data after exposing the cells with 1 µg/ml or 10 µg/ml of ZnO-1 or TiO_2 for 6 or 24 h in three replicate experiments (see **Figure S4** and **Table S3**). The subclusters separating different treatments are highlighted. The gene expression data for the differentially expressed genes is shown in **Table S9**.
