# Supplementary material for: Gene Expression Profiling of Immune-Competent Human Cells Exposed to Engineered Zinc Oxide or Titanium Dioxide Nanoparticles
Source: PLoS One. 2013 Jul 22;8(7):e68415. doi: 10.1371/journal.pone.0068415 (PMC3718780; doi:10.1371/journal.pone.0068415)
Supplement: Methods S1 — Supplementary methods containing nanoparticle production and characterization, and cell viability analysis methods. (DOCX) [file pone.0068415.s024.docx]

**Gene Expression Profiling of Immune-Competent Human Cells Exposed to Engineered Zinc Oxide or Titanium Dioxide Nanoparticles**

Soile Tuomela^1,2^, Reija Autio^3^, Tina Buerki-Thurnherr^4^, Osman Arslan^5^, Andrea Kunzmann^6^, Britta Andersson-Willman^7^, Peter Wick^4^, Sanjay Mathur^7^, Annika Scheynius^7^, Harald F. Krug^4^, Bengt Fadeel^6^, and Riitta Lahesmaa^1,*^

*^1^Turku Centre for Biotechnology, University of Turku and Åbo Akademi University, Turku, Finland;*

*^2^Turku Doctoral Programme of Biomedical Sciences, Turku, Finland;*

*^3^Department of Signal Processing, Tampere University of Technology, Tampere, Finland;*

*^4^Swiss Federal Laboratories for Material Science and Technology, Laboratory for Materials-Biology Interactions, St. Gallen, Switzerland;*

*^5^Inorganic and Materials Chemistry, University of Cologne, Cologne, Germany*

*^6^Division of Molecular Toxicology, Institute of Environmental Medicine, Karolinska Institutet, Stockholm, Sweden;*

*^7^Translational Immunology Unit, Department of Medicine Solna, Karolinska Institutet and University Hospital, Stockholm, Sweden;*

*^*^Corresponding author*

Correspondence: Riitta Lahesmaa, Turku Centre for Biotechnology, University of Turku and Åbo Akademi University, P.O. Box 123, FIN-20521 Turku, Finland, Tel: +358 2 333 8601, Fax: +358 2 251 8808, email: riitta.lahesmaa@btk.fi

**SUPPLEMENTARY METHODS**

**Nanoparticle production**

The synthesis and characterization of ZnO-2, ZnO-3 and ZnO-4 particles was recently reported by us [1]. The physico-chemical properties of the particles, ZnO-5 (microwave synthesis, diethylene glycol modified), ZnO-6 (non-hydrolytic thermal decomposition of the precursor, mandelic acid modified), ZnO-7 (phase-transfer synthesis, gluconic acid modified), ZnO-8 (non-hydrolytic thermal decomposition of the precursor, citric acid modified) and ZnO-9 (non-hydrolytic thermal decomposition of the precursor, folic acid modified), are assembled in  **Table S1** and **Table S2**.

**ZnO-5:** 40 ml diethylene glycol (DEG) was added to Zn(CH_3_COO)_2_·2H_2_O precursor (0.22 g; 0.001 mol) and stirred at 70 °C for 3 h for a homogeneous mixture. 6 ml of this solution was placed into a 10 ml microwave glass vessel, secured with a Teflon cap and microwave reaction was carried out at 200 °C, for 15 min (300 W) under magnetic stirring. Particles were centrifuged and washed with H_2_O and EtOH. Particles were dried in an oven at 80 °C and stored for testing.

**ZnO-6:** For the synthesis of the mandelic acid modified nanoparticles, 1.54 g (1.7 mmol) Zn(Oleate)_2_ precursor [2] was mixed with 17.2 ml (0.055 mol) %85 oleylamine and 9.7 ml (0.030 mol) oleic acid in a three-necked flask. This mixture was heated with 5 °C/min range using a thermocouple under an argon atmosphere until 285-290 °C. Solution was kept for 1 h at this temperature. After 1 h refluxing time, thermocouple was removed and the mixture was brought to room temperature. EtOH was added to precipitate and wash the nanoparticles followed by drying under vacuum. For the surface modification of dried particles, 500 mg of particles were placed into a beaker and 30 ml toluene was added. This mixture was treated with an ultrasonic finger for 30 min in order to get homogeneous suspension. 300 mg of mandelic acid was dissolved in 15 ml methanol separately in another beaker and added into the toluene-ZnO mixture within 30 s. This mixture was heated overnight at 65 °C and resulting nanoparticles were washed with MeOH and acetone respectively and finally dried in oven at 80 °C.

**ZnO-7:** 75 ml EtOH was added to 6.3 g Zn(Oleate)_2_ (0.01 mol) precursor and the mixture was ultrasonicated for 10 min followed by heating the solution to 80 °C in a rounded flask. In another flask 0.6 g NaOH (0.015 mol)/75 ml MeOH mixture was prepared and added into the Zn(Oleate)_2_-EtOH mixture within 30 s. Reaction mixture was refluxed at 80 °C under nitrogen gas flow about 72 h. 150 ml hexane was added at the end of the reflux treatment and the mixture was ultrasonicated. After centrifugation, particles were washed with hexane and water and acetone, respectively. 500 mg of dried particles were dissolved in 30 ml CHCl_3_ and treated ultrasonically for 10 min and mixed with 300 mg of gluconic acid in an aqueous MeOH (50/50) mixture followed by refluxing for 1 h at 60 °C. Solvents were removed by centrifugation and gluconic acid modified nanoparticles were washed with an EtOH, water and aceton respectively and dried under vacum.

**ZnO-8:** For the synthesis of the citric acid modified ZnO nanoparticles, 5.00 g (8.0 mmol) Zn(Oleate)_2_ precursor was mixed with 3 ml (0.008 mol) oleic acid and 14.6 ml (0.040 mol) from %85 oleylamine in a three-necked flask. Mixture was heated with a thermocouple under an argon atmosphere until 300 °C. After keeping 1 h at the same temperature solution was cooled down to room temperature. EtOH was added to precipitate and wash the nanoparticles. Particles were cleaned with acetone and ethanol and dried under vacuum. For the surface modification procedure, 500 mg ZnO nanoparticles were dispersed in toluene by ultrasonication. In another flask, 250 mg of citric acid was dissolved in 20 ml methanol and added into the toluene-ZnO mixture within 30 s followed by refluxing at 65 °C overnight. Obtained nanoparticles were washed with MeOH and acetone and dried at 60 °C.

**ZnO-9:** To 5.00 g Zn(CH_3_COO)_2_.2H_2_O (0.023 mol), oleylamine (6.25 ml; 0.016 mol) was added in a three-necked flask. Mixture was heated up to 130 °C under nitrogen atmosphere and maintained for 45 min for degassing the reaction mixture. After cooling down to room temperature 25 ml EtOH was added for washing and precipitate the particles. Cleaned and dried particles (500 mg) were dispersed in 30 ml toluene. In another flask, 150 mg of folic acid was dissolved in 10 ml MeOH and added to the toluene-ZnO mixture in 30 s. This mixture was ultrasonicated for 5 min and refluxed at 70 °C overnight and the resulting particles were washed with MeOH and acetone followed by a drying step.

**Nanoparticle characterization**

Transmission electron microscopy (TEM) investigations were carried out on a LEO 912 Omega (Zeiss, Oberkochen, Germany) (**Figure S1).** The instrument was operated at 120 kV with zero-loss conditions excluding inelastically scattered electrons to achieve a higher imaging contrast. Size distribution charts were acquired by counting 100 items for each sample and plotting with respect to their frequencies. Non-linear fitting on the obtained particle sizes resulted an average value with a calculated statistical standart deviation for each sample (**Figure S1**). Representative TEM images for each EN is shown in **Figure S2**. Zeta potentials and hydrodynamic light scattering (DLS) of the nanoparticle dispersions were measured using a Zetasizer Nano ZS (Malvern Instruments, UK). For the measurements, the nanoparticle suspensions (1 mg/ml) were prepared in deionised water or RPMI-medium and were sonicated for 3 min using an ultrasonic probe (70W, 20 kHz, Ultrasonic Homogenizer Bandelin Sonopuls UV 70). For DLS experiments, stock suspensions were further diluted to 100 ng/ml of ZnO in H_2_O or RPMI-medium and immediately measured at 25 °C on the Zetasizer Nano ZS at a detection angle of 173° and a 3 mW He-Ne laser operating at a wavelength of 633 nm. Powder X-ray diffraction (XRD) patterns of the ENs were measured with a STOE-STADI MP vertical system in transmission mode using Cu Kα (α=0.15406 nm) radiation **Figure S3**). Fourier transform infrared (FT-IR) analyses were carried out with PerkinElmer Spectrum 400 with Universal ATR Sampling Accessory in the 400-4000 cm^-1^ range. Thermogravimetric analysis (TGA) of the obtained particles was done in the temperature range from 30 °C to 800 °C with a heating rate of 10 °C/min under nitrogen atmosphere (flow rate 25 ml/min) using Mettler Toledo TGA/DSC 1 Star^e^ system. XRD, TGA and FT-IR data is presented in **Table S1** or published by Buerki-Thurnherr *et al*. [1]. DLS, PDI, and Zeta potential measurements were done both immediately and after 3 h of storage of the ZnO dispersions. The dispersion were ultrasonicated for 2 min before the measurement (70 W, 20 kHz, Ultrasonic Homogenizer Bandelin Sonopuls UV 70) (**Table S2**).

**
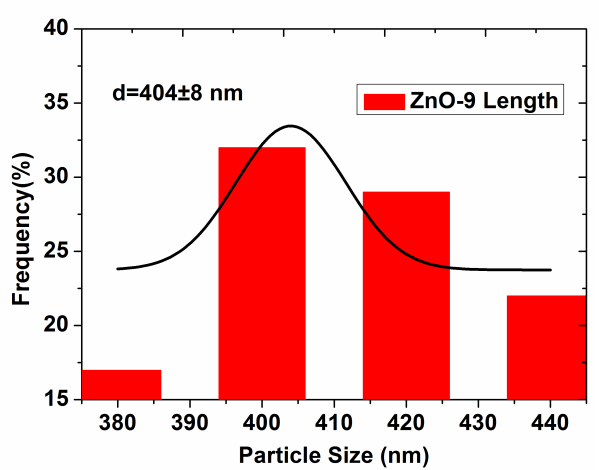
**

**Cell viability analysis**

Mitochondrial function in HMDM as a marker for cell viability was determined by using 3-(4,5-dimethyldiazol-2-yl)-2,5 diphenyl-tetrazolium bromide (MTT) (Sigma Aldrich). Monocytes were seeded into a 96-well plate and differentiated to HMDM as described previously [3]. Cells were exposed to nanoparticles at the indicated concentrations for 24 and 48 h. After exposure, the supernatant was removed and cells were washed once with phosphate-buffered saline (PBS) (pH 7.4). 100 μl of MTT solution (0.5 mg/ml) was added and incubated for 3 h at 37 °C. Finally, 50 μl of dimethyl sulfoxide (DMSO) (Sigma Aldrich) was added to dissolve the formazan crystals. MTT conversion was quantified by measuring the absorbance at 570 nm using a spectrophotometer (Infinite F200, Tecan, Männedorf, Switzerland)

The protocol for propidium iodide (PI) and Annexin V (AV) staining, and the viability data for ZnO-1 to ZnO-4 is reported in Buerki-Thurnherr *et at*. [1]. Fas ligand (fas) was used as a positive control in the analysis.

REFERENCES

1. Buerki-Thurnherr T, Xiao L, Diener L, Arslan O, Hirsch C, et al. (2013) In vitro mechanistic study towards a better understanding of ZnO nanoparticle toxicity. Nanotoxicology 7:402-416.

2. Choi SH, Kim EG, Park J, An K, Lee N, et al. (2005) Large-scale synthesis of hexagonal pyramid-shaped ZnO nanocrystals from thermolysis of zn-oleate complex. J Phys Chem B 109: 14792-14794.

3. Kunzmann A, Andersson B, Vogt C, Feliu N, Ye F, et al. (2011) Efficient internalization of silica-coated iron oxide nanoparticles of different sizes by primary human macrophages and dendritic cells. Toxicol Appl Pharmacol 253: 81-93.
